# Preoperative contrast-enhanced CT prediction of distinct vascular patterns in solitary early-stage hepatocellular carcinoma and its prognostic value

**DOI:** 10.1186/s13244-026-02224-5

**Published:** 2026-02-16

**Authors:** Wanli Zhang, Wen Lv, Yi Long, Jiaxin Lin, Jiamin Li, Chuanxian Zhang, Yandong Zhao, Jie Zhan, Shengsheng Lai, Mingyong Gao, Xinqing Jiang, Ruimeng Yang

**Affiliations:** 1 Department of Radiology, Guangzhou First People’s Hospital, Guangzhou, China; 2 Department of Radiology, Jinan University First Affiliated Hospital, Guangzhou, China; 3 Department of Radiology, The Zhaoqing Hospital of the Third Affiliated Hospital, Sun Yat-sen University, Zhaoqing, China; 4 Department of Radiology, The First Affiliated Hospital of Nanchang University, Nanchang, China; 5 School of Medical Equipment, Guangdong Food and Drug Vocational College, Guangzhou, China; 6 Department of Radiology, The First People’s Hospital of Foshan, Foshan, China

**Keywords:** Hepatocellular carcinoma, Microvascular invasion, Vessels that encapsulate tumor clusters, Computed tomography, Recurrence-free survival

## Abstract

**Objectives:**

To investigate the value of qualitative and quantitative contrast-enhanced CT (CECT) features for noninvasive identification of two distinct vascular patterns, vessels that encapsulate tumor clusters (VETC) and/or microvascular invasion (MVI), in solitary early-stage (BCLC 0-A) hepatocellular carcinoma (HCC) and assess their prognostic implications.

**Materials and methods:**

We retrospectively included 347 patients with solitary early-stage HCC who underwent preoperative CECT and subsequent resection at two centers. Patients were divided into V/M+ (MVI and/or VETC positive, *n* = 174) and VM− (both MVI and VETC negative, *n* = 173) groups based on histopathology. Four predictive models (clinical, CT quantitative, CT qualitative, and combined) integrating clinical and CECT features were developed and validated for identifying V/M+ status. The optimal model was further applied to predict 2-year recurrence-free survival (RFS). Sensitivity analysis was performed using propensity score matching (PSM). Models’ performance was evaluated and compared using AUC analyses and DeLong tests.

**Results:**

The combined model [serum AFP ≥ 200 ng/mL, non-smooth tumor margin, internal arteries, and lower tumor-to-liver density ratio in the portal venous phase (P-TLR)] achieved optimal predictive performance for V/M + HCC, with training AUC of 0.784 and 0.782 pre- and post-PSM, and external validating AUC of 0.794. A derived V/M+ score stratified patients, with higher scores associated with significantly shorter 2-year RFS. V/M+ score ≥ 34 and tumor size ≥ 60 mm were significant predictors of HCC recurrence (*p* < 0.05).

**Conclusion:**

The combined model integrating clinical and CECT-based features, enables non-invasive assessment of V/M status in early-stage solitary HCC and effectively stratifies patients according to recurrence risk.

**Critical relevance statement:**

Specific CT-based qualitative and quantitative features are associated with a distinct vascular pattern of BCLC stage 0-A HCC. The developed combined model and derived V/M+ score offer a reliable tool for clinicians to predict V/M + HCC and patients’ 2-year RFS.

**Key Points:**

- Specific CECT-based qualitative and quantitative features are associated with V/M + HCC at the BCLC stage 0-A.
- The developed combined model offers a reliable tool for clinicians to identify V/M + HCC.
- The derived V/M+ score helps stratify HCC patients into high- and low-risk groups for 2-year RFS, facilitating personalized management of HCC.

**Graphical Abstract:**

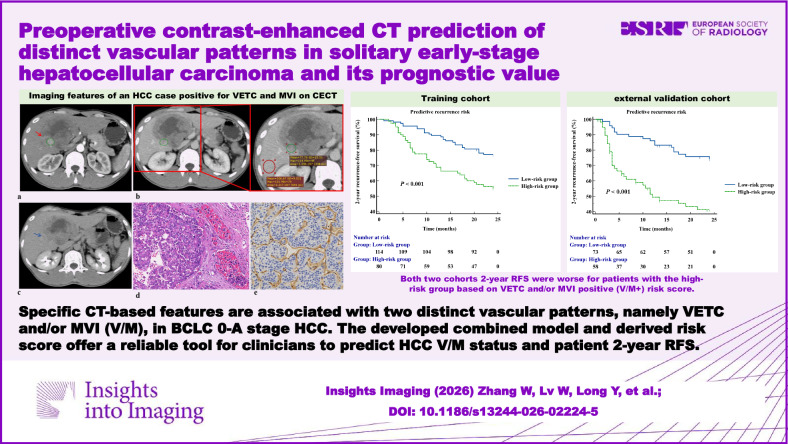

## Introduction

Hepatocellular carcinoma (HCC) remains a leading cause of cancer-related mortality worldwide, with unsatisfactory long-term outcomes even among patients receiving curative-intent therapies [1, 2]. The Barcelona Clinic Liver Cancer (BCLC) staging system is the global standard for HCC staging and treatment guidance in clinical management [3, 4]. Patients with BCLC stage 0-A HCC, known as early-stage HCC, typically present with a solitary nodule with well-preserved liver function, and lack vascular invasion or distant metastasis, making them ideal candidates for curative treatments [5, 6]. However, even in early-stage HCC, poor outcomes were observed [7], potentially due to the aggressive microvasculature characteristics of the tumor. The abnormal microvasculature of HCC raises the risk of hematogenous metastases [8]. Two different vascular patterns in HCC, including microvascular invasion (MVI) and vessels that encapsulate tumor clusters (VETC), have been reported to potentially worsen patient’s prognosis [9].

MVI has been recognized as a major predictor of recurrence and poor prognosis following curative treatments [10]. The incidence of MVI increases with tumor size [7]. In addition to MVI, VETC has emerged as another distinctive vascular pattern associated with aggressive tumor behavior in HCC. Unlike traditional hematogenous metastasis driven by epithelial-mesenchymal transition (EMT), VETC-mediated metastasis involves a continuous vascular network encapsulating tumor clusters, facilitating dissemination independent of EMT processes [11, 12]. These vascular structures facilitate direct entry of tumor clusters into circulation through anastomoses with peritumoral vessels [11]. Recent transcriptomic analyses revealed that VETC-positive (VETC+) HCCs exhibited diminished immune cell activation compared to VETC-negative (VETC−) HCCs [13, 14]. A study demonstrated that Sorafenib markedly extended survival in VETC + HCC patients, but not in those with the VETC- pattern [15]. Thus, predicting different vascular patterns in HCC pretreatment is crucial for personalized treatment and optimizing outcomes in HCC patients.

Moreover, MVI and VETC represented distinct yet complementary vascular invasion mechanisms. HCC patients with VETC+ and/or MVI-positive (MVI+) (termed V/M+) exhibited worse prognosis than those with both VETC- and MVI-negative (MVI−) (termed VM−) pattern [9]. Therefore, comprehensive pretherapeutic assessment of both vascular phenotypes is essential. However, accurate characterization remains challenging, particularly because HCC diagnosis is often established solely on typical imaging features without histopathological confirmation [3, 16, 17]. In this context, preoperative imaging holds significant potential for estimating MVI and/or VETC status. Several studies have identified specific imaging features associated with MVI+ or VETC + HCC, such as non-smooth tumor margin, arterial peritumoral enhancement, intratumoral necrosis or severe ischemia, etc [18–23]. Additionally, quantitative magnetic resonance imaging (MRI) features have been shown to correlate with VETC + HCC [23, 24]. To date, various predictive models based on MRI for VETC+, MVI+, or V/M + HCC have been defined [22–25]. However, the imaging features of contrast-enhanced computed tomography (CECT), especially quantitative features related to V/M + HCC at the early stage, remain unclear.

Therefore, we aimed to develop and externally validate a diagnostic model for the MVI+ and/or VETC+ (V/M+) pattern of BCLC stage 0-A solitary HCC by using CECT qualitative and quantitative features, and to investigate the model’s utility in predicting 2-year recurrence-free survival (RFS) of HCC patients.

## Materials and methods

### Patient information

**Fig. 1:**
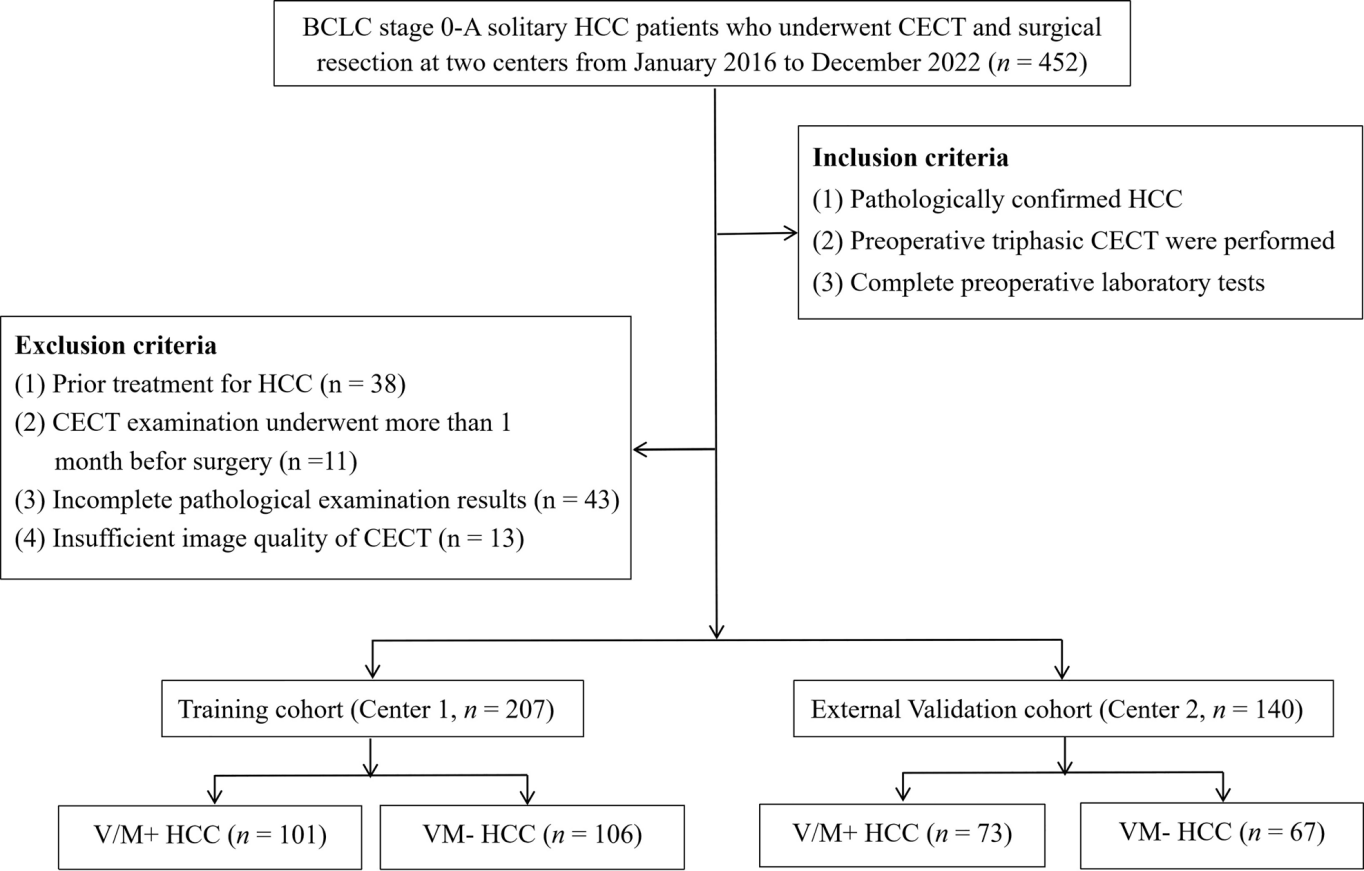
Flowchart of study population with inclusion and exclusion criteria. CECT, contrast-enhanced computed tomography; HCC, hepatocellular carcinoma; BCLC, Barcelona clinic liver cancer; V/M, VETC and/or MVI

This study was approved by the Institutional Review Board of the First People’s Hospital of Foshan (Center 1) and Guangzhou First People’s Hospital (Center 2). Informed consent was waived due to the study’s retrospective nature (ethical approval ID: 2022-145-03). Consecutive adult patients (≥ 18 years) who underwent preoperative triphasic CECT followed by hepatic resection for HCC between January 2016 and December 2022 were enrolled. The inclusion criteria were as follows: (1) histopathologically confirmed solitary HCC with BCLC stage 0 or A; (2) availability of preoperative triphasic CECT, and (3) complete preoperative laboratory data. Patients were excluded if they met any of the following criteria: (1) receipt of prior anticancer therapy, such as transcatheter arterial chemoembolization, radiofrequency ablation, or systemic chemotherapy; (2) an interval exceeding one month between CECT and surgery; (3) incomplete pathological data, including missing assessment of MVI or VETC status; or (4) poor-quality CECT images. Finally, a total of 207 patients from Center 1 were assigned to the training cohort, and 140 patients from Center 2 were allocated to the external validation cohort. Figure 1 showed a schematic workflow of this study.

Clinical variables included age, sex, hepatic B or C infection status, and laboratory indicators: serum Alpha-fetoprotein (AFP) level, red blood cell (RBC) count, white blood cell (WBC) count, hemoglobin, platelet (PLT) count, neutrophil count, lymphocyte count, serum albumin, aspartate aminotransferase (AST), alanine aminotransferase (ALT), and γ-glutamyl transpeptidase (GGT) levels. Derived ratios were calculated, including the platelet-to-white blood cell ratio (PWR), platelet-to-lymphocyte ratio (PLR), neutrophil-to-lymphocyte ratio (NLR), GGT-to-lymphocyte ratio (GLR), aspartate aminotransferase-to-alanine aminotransferase ratio (AAR), and γ-glutamyl transpeptidase-to-aspartate aminotransferase ratio (GAR). All clinical and laboratory data were obtained within one week prior to surgery.

Postoperative follow-up examinations for patients were conducted using abdominal ultrasound, CECT, or MRI every 3-6 months. According to imaging or pathological findings, 2-year RFS was recorded, determined as the time from surgery to the initial-documented tumor recurrence (whether intrahepatic or distant), all-cause death, or the last follow-up (December 31, 2024) if censored.

### CECT image acquisition and analysis

Preoperative CECT was performed using multiple scanners and included three enhancing phases: arterial phase (AP) at 30–40 s, portal venous phase (PVP) at 60–90 s, and delayed phase (DP) at 120–250 s. Detailed CECT parameters are outlined in Appendix S1.

Two board-certified radiologists (W.Z. and R.Y., with 7 and 20 years of experience in abdominal imaging, respectively) independently reviewed all CECT images. They were blinded to all patient details except for the HCC diagnosis and were responsible for assessing both qualitative and quantitative features. Qualitative features were assessed using the 2018 Liver Imaging Reporting and Data System (LI-RADS) major and auxiliary features [26], as well as previously reported key features. Any disagreements were resolved by a senior radiologist. Quantitative features included: (1–3) the tumor-to-liver density ratio (TLR), (4–6) the tumor-to-aorta density ratio (TAR), and (7–9) the standardized tumor-to-aorta density ratio (STAR) in the AP, PVP, and DP, respectively. Each reviewer independently measured all quantitative features three times, and the average of these measurements were calculated as the final results. Appendix S2 and Table S1 provided descriptions of the measurement methods and all CECT image features.

### Histopathologic analysis

An experienced pathologist (Y.Z., with 15 years of experience in abdominal pathology), who was blinded to the clinical and imaging data, reviewed all surgical specimens. Tumor characteristics, including number of tumors, Edmondson-Steiner (ES) grade, presence of cirrhosis, and vascular patterns (VETC and MVI) were recorded. VETC+ was defined as sinusoid-like vessels forming web-like networks and encapsulating individual tumor clusters in more than 5% of the tumor area, as detected by CD34 immunostaining. MV+I was defined as tumor cell nests in an endothelial cell-lined vascular lumen, including blood vessels within the capsule, predominantly observed in portal vein branches under microscopic examination. The V/M+ pattern was defined as VETC+ and/or MVI+, while the VM− pattern was defined as both VETC− and MVI−.

### Development and validation of the V/M + HCC predictive model and the V/M+ score

Univariable and multivariable logistic regression with forward stepwise maximum likelihood estimation was conducted to identify independent predictors of V/M + HCC among all features in the training cohort, avoiding multicollinearity (variance inflation factor < 5), with a significance level of *p* < 0.05 for variable entry. Four predictive models were established: clinical, CT quantitative, CT qualitative, and combined models utilizing clinical factors, CT quantitative measurements, CT qualitative features, and all statistically significant indicators from the aforementioned three models, respectively. Each model was conducted using the 1000 bootstrap resampling method in the training cohort and externally validated. A CT scanner-based stratification analysis was conducted. Optimal cut-off values for significant CECT quantitative parameters were determined using receiver operating characteristic (ROC) analysis by maximizing the Youden index. Rebuilt the best model with continuous candidate variables due to methodological considerations as a sensitivity analysis. A simplified and streamlined nomogram was developed based on the most effective prediction model.

According to the independent variables from the optimal predictive model, each patient was assigned a score for 2-year RFS, named the V/M+ score. This score was weighted by the respective β coefficients, with the largest β coefficient scaled as 20 points and the others proportionally rounded to the nearest integers to improve clinical utility. The optimal cut-off, determined by maximizing the Youden index in the training cohort, divided individuals into high- or low-risk groups for recurrence.

Additionally, propensity score matching (PSM) was performed as a sensitivity analysis to reduce the potential confounding effects and selection bias in this multicenter study (Appendix S3). The four predictive models and V/M+ score were also tested in the PSM-adjusted cohorts in the training cohort to investigate whether their performance remained stable even under a scenario where major baseline confounders between centers were artificially controlled.

### Statistical analysis

The Student’s *t*-test or Mann–Whitney *U*-test was used to compare continuous variables, while the χ test or Fisher’s Exact test was applied to compare categorical variables. The area under the curve (AUC) through ROC analysis, accuracy (ACC), sensitivity  (SEN), specificity (SPE), positive predictive value (PPV), and negative predictive value (NPV) were employed to evaluate the four predictive models’ performance, with the DeLong test used to compare differences between models. The interobserver agreement on CECT findings was evaluated using either Cohen’s kappa coefficient or the intraclass correlation coefficient (ICC). Two-year RFS was evaluated using Kaplan–Meier analysis and compared with the log-rank test. Univariable and multivariable Cox Proportional Hazards regression analyses with Schoenfeld residuals were conducted on the significant features in the univariable logistic regression and V/M+ score to determine prognostic factors.

Statistical analyses were performed using SPSS 25.0 software (IBM SPSS Corporation), R software (version 4.2.2), and MSTATA software (www.mstata.com). A two-sided *p* < 0.05 was considered statistically significant.

## Results

### Clinical and pathological characteristics

Patient characteristics are summarized in Table 1. Briefly, 347 patients were included in this study: 207 HCC patients in the training cohort (173 men, 101 [48.79%] V/M+ HCCs) and 140 patients in the external validation cohort (118 men, 73 [52.14%] V/M+ HCCs). Immunohistochemistry results showed that 72 (34.78%) lesions in the training cohort were VETC+ and 64 (30.92%) were MVI + ; in the external validation cohort, 45 (32.14%) were VETC+ and 64 (45.71%) were MVI+. V/M+ HCC exhibited a higher ES grade (*p* < 0.05). Patients with V/M+ HCC were younger and had higher AFP levels compared to VM- HCC patients (all *p* < 0.05).

**Table 1:** Demographic and clinical characteristics of HCC patients

	Training cohort (*n* = 207)			External validation cohort (*n *= 140)			
	V/M+ HCC (*n* = 101)	VM- HCC (*n* = 106)	*p*	V/M+ HCC (*n* = 73)	VM- HCC (*n* = 67)	*p*	*p*^*@*^
Age (year)			0.007^*^			0.063^*^	0.414^*^
< 50	38 (37.60)	22 (20.80)		23 (31.51)	12 (17.91)		
≥ 50	63 (62.40)	84 (79.20)		50 (68.49)	55 (82.09)		
Sex			0.551^*^			0.251^*^	0.860^*^
Male	86 (85.10)	87 (82.10)		64 (87.67)	54 (80.60)		
Female	15 (14.90)	19 (17.90)		9 (12.33)	13 (19.40)		
Hepatic virus infection			0.032^*^			0.001^*^	0.950^*^
Present	88 (87.10)	80 (75.50)		67 (91.78)	47 (70.15)		
Absent	13 (12.90)	26 (24.50)		6 (8.22)	20 (29.851)		
AFP (ng/mL)			0.001^*^			< 0.001^*^	0.292^*^
≥ 200	44 (43.60)	23 (21.70)		40 (54.80)	13 (19.40)		
< 200	57 (56.40)	83 (78.30)		33 (45.20)	54 (80.60)		
WBC (10^9^/L)	6.22 (5.20, 7.54)	5.96 (4.90, 7.37)	0.099^b^	6.57 (5.66, 8.30)	6.250 (5.14, 7.38)	0.151^b^	0.108^b^
RBC (10^12^/L)	4.57 (4.17, 4.93)	4.59 (4.10, 5.04)	0.969^a^	4.73 (4.25, 5.15)	4.39 (4.08, 4.72)	**0.022**^b^	0.816^b^
PLT (10^9^/L)	204.00 (155.50, 254.00)	191.50 (158.00, 225.75)	0.261^b^	195.00 (148.00, 260.00)	190.00 (150.00, 248.50)	0.570^b^	0.927^b^
Hemoglobin (g/L)	137.00 (126.50, 149.00)	134.00 (119.00, 149.25)	0.322^b^	141.00 (120.00, 153.00)	134.00 (121.50, 142.50)	0.284^b^	0.990^b^
Neutrophil (10^9^/L)	3.71 (3.06, 4.98)	3.54 (2.70, 4.51)	0.210^b^	3.95 (3.25, 5.12)	3.54 (2.74, 4.61)	0.160^b^	0.384^b^
Lymphocyte (10^9^/L)	1.57 (1.22, 1.90)	1.56 (1.23, 1.95)	0.766^a^	1.60 (1.18, 2.04)	1.63 (1.18, 2.08)	0.948^b^	0.610^b^
PWR	30.96 (24.92, 39.26)	30.93 (23.10, 39.77)	0.551^b^	32.09 (23.20, 37.26)	30.45 (23.80, 39.07)	0.723^b^	0.418^b^
PLR	132.92 (97.57, 167.88)	114.29 (95.38, 159.32)	0.287^b^	118.87 (100.00, 180.00)	115.33 (90.12, 153.21)	0.405^b^	0.716^b^
NLR	2.50 (1.73, 3.24)	2.20 (1.73, 3.13)	0.262^b^	2.66 (1.90, 3.29)	2.07 (1.57, 3.23)	0.078^b^	0.866^b^
GLR	36.99 (20.72, 66.22)	34.02 (19.11, 56.71)	0.508^b^	46.77 (20.69, 97.39)	40.00 (23.96, 84.97)	0.667^b^	**0.028**^b^
ALT (U/L)	30.00 (22.00, 45.00)	29.00 (19.00, 39.25)	0.383^b^	32.00 (23.00, 48.00)	25.00 (18.00, 40.00)	**0.037**^b^	0.673^b^
AST (U/L)	33.00 (25.50, 48.00)	30.50 (23.00, 40.00)	0.104^b^	41.00 (29.00, 65.00)	33.00 (27.00, 45.50)	**0.023**^b^	**0.004**^b^
GGT (U/L)	56.00 (31.00, 89.50)	49.00 (29.75, 79.25)	0.380^b^	71.00 (33.00, 159.00)	60.00 (40.50, 99.00)	0.617^b^	**0.007**^b^
AAR	1.13 (0.83, 1.43)	1.03 (0.83, 1.27)	0.142^b^	1.09 (0.97, 1.52)	1.17 (0.96, 1.54)	0.746^b^	**0.006**^b^
GAR	1.58 (1.08, 2.22)	1.61 (1.00, 2.95)	0.807^b^	1.58 (1.00, 2.92)	1.78 (1.12, 3.33)	0.318^b^	0.379^b^
Albumin (g/L)	39.80 (37.70, 41.85)	40.80 (38.40, 43.00)	0.240^a^	37.90 (35.10, 41.80)	37.30 (34.90, 40.45)	0.337^b^	**< 0.001**^b^
ES grade			< 0.001^*^			< 0.001^*^	0.003^*^
I–II	16 (15.80)	54 (50.90)		21 (28.77)	49 (73.13)		
III–IV	85 (84.20)	52 (49.10)		52 (71.23)	18 (26.87)		
VETC			< 0.001^*^			< 0.001^*^	0.540^*^
Present	72 (71.30)	0 (0.00)		45 (61.64)	0 (0.00)		
Absent	29 (28.70)	106 (100.00)		28 (38.36)	67 (100.00)		
MVI			< 0.001^*^			< 0.001^*^	0.005^*^
Present	64 (63.40)	0 (0.00)		64 (87.67)	0 (0.00)		
Absent	37 (36.60)	106 (100.00)		9 (12.33)	67 (100.00)		
Cirrhosis			0.480^*^			0.877^*^	0.081^*^
Present	42 (41.60)	39 (36.80)		35 (47.95)	33 (49.25)		
Absent	59 (58.40)	67 (63.20)		38 (52.05)	34 (50.75)		

Unless indicated otherwise, data are the number of tumors, with percentages in parentheses

A *p* value less than 0.05 was considered statistically significant, presented in bold

*AFP* alpha-fetoprotein, *WBC* white blood cell, *RBC* red blood cell, *PLT* platelet, *ALT* alanine aminotransferase, *AST* aspartate aminotransferase, *GGT* γ-glutamyl transpeptidase, *PLR* PLT-to-lymphocyte ratio, *PWR* PLT-to-WBC ratio, *GLR* GGT-to-lymphocyte ratio, *NLR* neutrophil-to-lymphocyte ratio, *AAR* AST-to-ALT ratio, *GAR* GGT-to-AST ratio, *ES grade* edmondson-steiner grade, *V/M* VETC and/or MVI

^*^ Chi-square test

^a^ Student’s *t*-test. Data are median (interquartile range, IQR)

^b^ Mann–Whitney *U*-test. Data are median (interquartile range, IQR)

^@^ Comparison of training cohort and external validation cohort

### CECT features

**Fig. 2:**
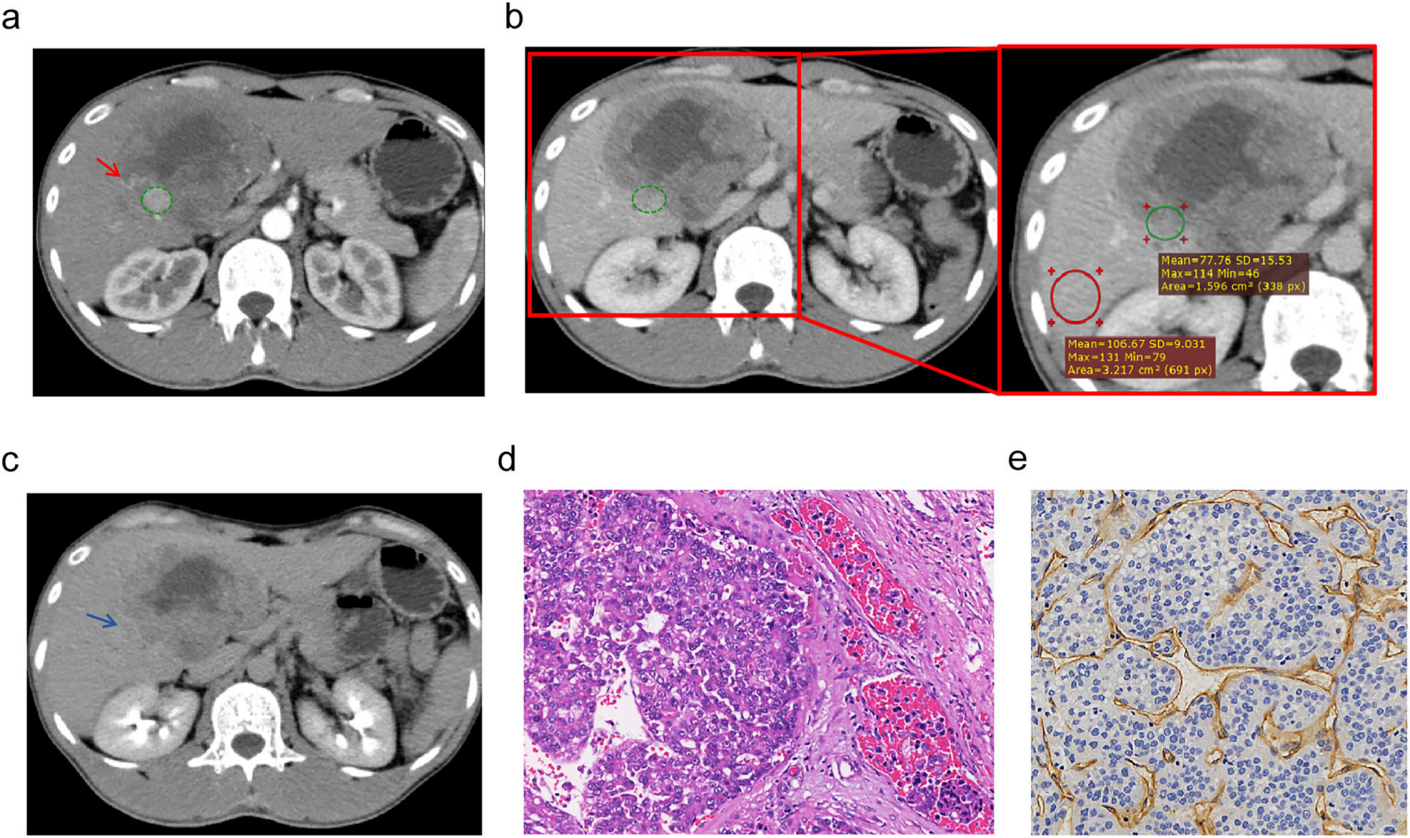
Imaging features of a V/M + HCC located at the junction of liver segments 4 and 5 in a 31-year-old man with a serum AFP level of 4643 ng/mL. The lesion was 74 mm in diameter, with internal arteries visible in the AP (**a**, red arrow), and featured a non-smooth tumor margin (**b**, **c**). The P-TLR of this tumor was measured as P-TLR = $\frac{{{\mathrm{Tumor}}}}{{{\mathrm{Liver}}}}$ = $\frac{77.76}{106.67}$ = 0.729 < 0.86 in the PVP (**b**). The tumor was classified as MVI+ on hematoxylin-eosin stain (**d**, ×400 magnification) and VETC+ on immunohistochemistry (**e**, ×400 magnification). A green circle was used to define the region of interest (ROI) for lesion measurement, and a red circle was used to mark the liver parenchyma area. AFP, alpha-fetoprotein; PVP, portal venous phase; P-TLR, tumor-to-liver density ratio in portal venous phase. V/M, VETC and/or MVI

**Fig. 3:**
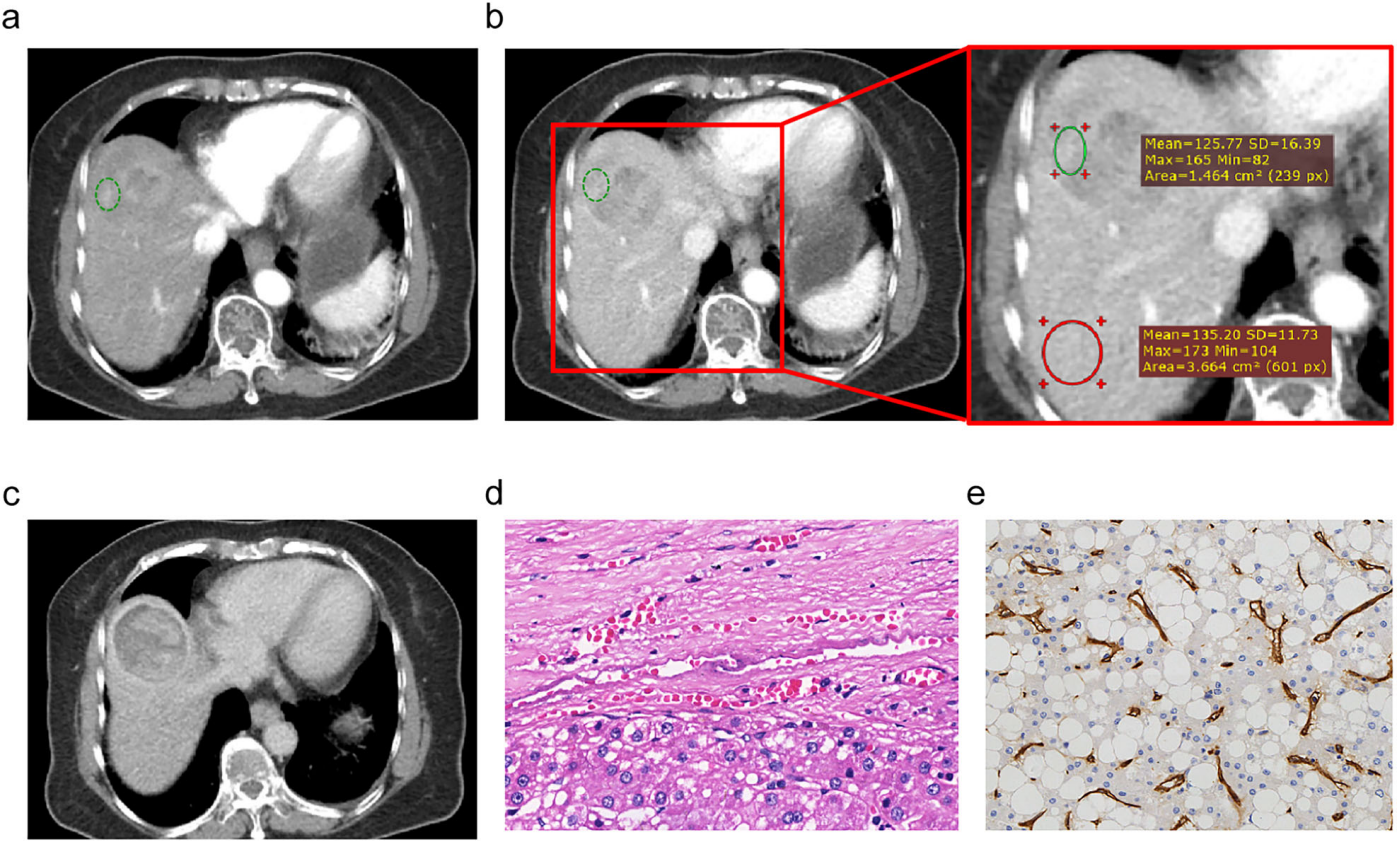
Imaging features of a VM- HCC in the liver segment 8 in an 86-year-old man with serum AFP level of 3.96 ng/mL. The lesion was 65 mm in diameter, without internal arteries in the AP (**a**). PVP and DP images shown a smooth tumor margin (**b**, **c**), The P-TLR of this tumor was measured as P-TLR = $\frac{{{\mathrm{Tumor}}}}{{{\mathrm{Liver}}}}$ = $\frac{125.77}{135.20}$ = 0.930 > 0.86 in the PVP (**b**). Tumor was classified as MVI- on hematoxylin-eosin stain (**d**, ×400 magnification) and VETC- on immunohistochemistry (**e**, ×400 magnification). A green circle was used to define the region of interest (ROI) for lesion measurement, and a red circle was used to mark the liver parenchyma area. AFP, alpha-fetoprotein; DP, delayed phase; PVP, portal venous phase; P-TLR, tumor-to-liver density ratio in portal venous phase. V/M, VETC and/or MVI

The qualitative CECT imaging features demonstrated moderate to excellent inter-observer agreement (Table S2), with Cohen’s kappa coefficients ranging from 0.681 (95% CI: 0.522–0.840) to 1.00 (95% CI: 1.000–1.000). The quantitative measurements showed similar performance, with ICC values ranging from 0.729 (95% CI: 0.607–0.818) to 0.972 (95% CI: 0.960–0.981). Compared to VM- HCCs, V/M+ HCCs were significantly more likely to present with: non-smooth tumor margin, intratumoral necrosis or severe ischemia, internal arteries, and two-trait predictor of venous invasion (TTPVI) (all *p* < 0.05, Table 2). Quantitative analyses revealed that V/M + HCC had notably lower values of TLR (P-TLR, D-TLR), TAR (P-TAR, D-TAR), and STAR (P-STAR, D-STAR) in both PVP and DP (all *p* < 0.05). Additionally, tumor size was significantly larger in V/M+ HCCs than in VM- HCCs (*p* < 0.05). Representative CECT images of V/M+ and VM- HCC were shown in Figs. 2 and 3.

**Table 2:** Comparison of CT qualitative and quantitative features between V/M+ HCC and VM- HCC

	Training cohort (*n* = 207)			External validation cohort (*n* = 140)		
	V/M+ HCC (*n* = 101)	VM- HCC (*n* = 106)	*p*	V/M+ HCC (*n* = 73)	VM- HCC (*n* = 67)	*p*
Qualitative features						
Tumor margin			0.001^*^			< 0.001^*^
Smooth	29 (27.70)	54 (50.90)		18 (24.66)	36 (53.73)	
Non-smooth	73 (72.30)	52 (49.10)		55 (75.34)	31 (46.27)	
Capsule			0.315^*^			0.261^*^
Incomplete or absent	47 (46.50)	42 (39.60)		45 (61.64)	35 (52.24)	
Complete	54 (53.50)	64 (60.40)		28 (38.36)	32 (47.76)	
Enhancing capsule			0.273^*^			0.070^*^
Present	72 (71.30)	68 (64.20)		56 (76.71)	42 (62.69)	
Absent	29 (28.70)	38 (35.80)		17 (23.29)	25 (37.31)	
Fat in mass			0.371^*^			0.426^#^
Present	3 (3.00)	7 (6.60)		2 (2.74)	4 (5.97)	
Absent	98 (97.00)	99 (93.40)		71 (97.26)	63 (94.03)	
Blood products in mass			0.311^*^			0.475^*^
Present	7 (6.90)	4 (3.80)		7 (9.59)	9 (13.43)	
Absent	94 (93.10)	102 (96.20)		66 (90.41)	58 (86.57)	
Mosaic architecture			0.104^*^			0.493^*^
Present	32 (31.70)	23 (21.70)		17 (23.29)	19 (28.36)	
Absent	69 (68.30)	83 (78.30)		56 (76.71)	48 (71.64)	
Intratumoral necrosis or severe ischemia (≥ 20%)			0.001^*^			0.011^*^
Present	67 (66.30)	46 (43.40)		36 (49.32)	19 (28.36)	
Absent	34 (33.70)	60 (56.60)		37 (50.68)	48 (71.64)	
Intratumoral necrosis or severe ischemia (≥ 50%)			0.004^*^			0.186^*^
Present	30 (29.70)	14 (13.20)		12 (16.44)	6 (8.95)	
Absent	71 (70.30)	92 (86.80)		61 (83.56)	61 (91.05)	
Hypodense halo			0.227^*^			0.498^*^
Present	23 (22.80)	32 (30.20)		12 (16.44)	14 (20.90)	
Absent	78 (77.20)	74 (69.80)		61 (83.56)	53 (79.10)	
Internal arteries			< 0.001^*^			< 0.001^*^
Present	77 (76.20)	53 (50.00)		50 (68.49)	25 (37.31)	
Absent	24 (23.80)	53 (50.00)		23 (31.51)	42 (62.69)	
TTPVI			0.001^*^			< 0.001^*^
Positive	55 (54.50)	33 (31.10)		41 (56.16)	19 (28.36)	
Negative	46 (45.50)	73 (68.90)		32 (43.84)	48 (71.64)	
Corona enhancement			0.021^*^			0.497^#^
Present	11 (10.90)	3 (2.80)		6 (8.22)	3 (4.48)	
Absent	90 (89.10)	103 (97.20)		67 (91.78)	64 (95.52)	
Fade			0.714^*^			0.013^*^
Present	18 (17.80)	21 (19.80)		7 (9.59)	17 (25.37)	
Absent	83 (82.20)	85 (80.20)		66 (90.41)	50 (74.63)	
Nodule-in-nodule architecture			0.386^*^			0.635^*^
Present	23 (22.80)	19 (17.90)		15 (20.55)	16 (23.88)	
Absent	78 (77.20)	87 (82.10)		58 (79.45)	51 (76.12)	
Targetoid sign			0.865^*^			0.370^*^
Present	20 (19.80)	20 (18.90)		20 (27.40)	14 (20.90)	
Absent	81 (80.20)	86 (81.10)		53 (72.60)	53 (79.10)	
Rim APHE			0.586^*^			0.477^*^
Present	15 (14.90)	13 (12.30)		13 (17.81)	9 (13.43)	
Absent	86 (85.10)	93 (87.70)		60 (82.19)	58 (86.57)	
Peripheral washout			0.405^*^			0.052^*^
Present	11 (10.90)	8 (7.50)		12 (16.44)	4 (5.97)	
Absent	90 (89.10)	98 (92.50)		61 (83.56)	63 (94.03)	
Perfusion alteration			0.773^*^			0.169^#^
Present	12 (11.90)	14 (13.20)		7 (9.59)	2 (2.99)	
Absent	89 (88.10)	92 (86.80)		66 (90.41)	65 (97.01)	
Quantitative features						
Tumor size (mm)	53.40 (35.50, 82.00)	42.10 (32.08, 59.13)	**0.005**^b^	60.10 (41.70, 91.60)	48.40 (34.30, 70.60)	**0.013**^**b**^
ROI (cm^2^)	2.65 (1.70, 4.46)	2.34 (1.58, 3.78)	0.198^b^	2.45 (1.74, 4.62)	3.14 (1.96, 4.12)	0.871^b^
A-TLR	1.28 (1.15, 1.53)	1.38 (1.21, 1.62)	0.095^b^	1.16 (0.97, 1.34)	1.21 (1.03, 1.43)	0.186^b^
A-TAR	0.25 (0.21, 0.31)	0.28 (0.21, 0.34)	**0.031**^b^	0.28 (0.24, 0.37)	0.31 (0.24, 0.37)	0.646^b^
A-STAR	0.05 (0.03, 0.10)	0.08 (0.04, 0.12)	**0.031**^b^	0.04 (-0.01, 0.08)	0.05 (0.01, 0.11)	0.174^b^
P-TLR	0.85 (0.78, 0.95)	0.92 (0.86, 1.03)	**0.000**^b^	0.83 (0.72, 0.91)	0.94 (0.83, 1.05)	**< 0.001**^b^
P-TAR	0.67 (0.60, 0.74)	0.74 (0.68, 0.80)	**0.000**^b^	0.63 (0.55, 0.74)	0.69 (0.61, 0.77)	**0.016**^a^
P-STAR	−0.12 (−0.18, −0.04)	−0.07 (−0.11, 0.02)	**0.000**^b^	−0.14 (−0.22, −0.07)	−0.04 (−0.14, 0.04)	**< 0.001**^a^
D-TLR	0.88 (0.78, 0.94)	0.94 (0.84, 1.01)	**0.000**^b^	0.86 (0.77, 0.89)	0.91 (0.83, 1.00)	**< 0.001**^b^
D-TAR	0.72 (0.67, 0.80)	0.75 (0.69, 0.84)	**0.009**^b^	0.74 (0.69, 0.79)	0.80 (0.74, 0.86)	**0.002**^b^
D-STAR	−0.10 (−0.19, −0.05)	−0.05 (−0.14, 0.01)	**0.001**^b^	−0.13 (−0.19, −0.09)	−0.08 (−0.15, 0.00)	**< 0.001**^b^

Unless indicated otherwise, data are the number of tumors, with percentages in parentheses

A *p* value less than 0.05 was considered statistically significant, presented in bold

*APHE* arterial phase hyperenhancement, *TTPVI* two-trait predictor of venous invasion, *ROI* region of interest, *A* arterial phase, *P* portal venous phase, *D* delayed phase, *TLR* tumor-to-liver density ratio, *TAR* tumor-to-aorta density ratio, *STAR* standardized tumor-to-aorta density ratio, *V/M* VETC and/or MVI

^*^ Chi-square test

^#^ Fisher’s Exact test

^a^ Student’s *t*-test. Data are median (interquartile range, IQR)

^b^ Mann–Whitney *U*-test. Data are median (interquartile range, IQR)

### V/M + HCC predictive model

**Fig. 4:**
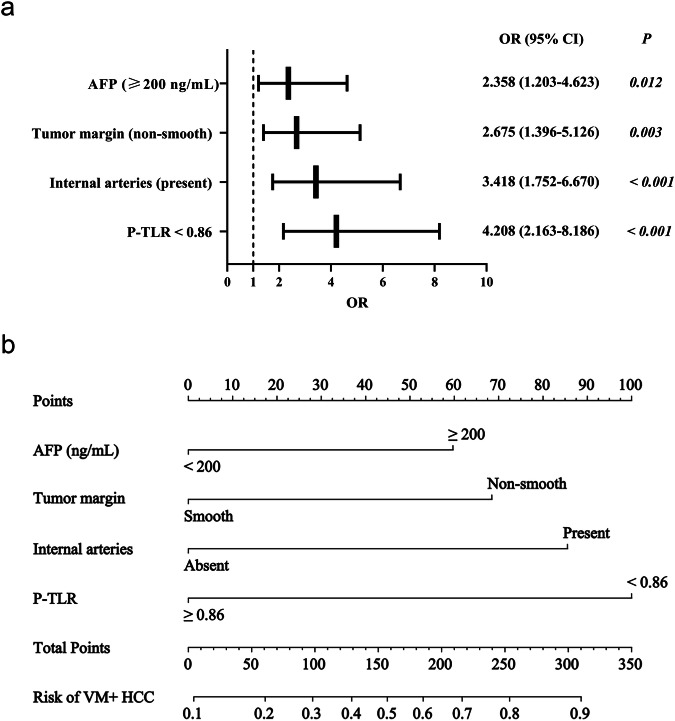
Forest plot (**a**) and nomogram (**b**) of independent predictors for V/M+ HCC in the combined model. The model presented a nomogram scaled according to the proportional regression coefficients of each predictor. The cumulative points from all variables on the bottom scale were converted into the probability of V/M+ HCC. AFP, alpha-fetoprotein; P-TLR, tumor-to-liver density ratio in portal venous phase; OR, Odds Ratio; CI, confidence interval; V/M, VETC and/or MVI

Table 3 and Fig. 4 demonstrated each model’s performance. In the clinical model, univariable and multivariable logistic regression analyses identified younger age (< 50 years old, odds ratio [OR], 1.943; *p* = 0.042) and elevated AFP level (≥ 200 ng/mL, OR, 2.476; *p* = 0.004) as independent predictors of V/M+ HCC (Table S3), with AUCs of 0.636 and 0.707 in the training and validation cohorts, respectively.

**Table 3:** Performances of the four models for predicting V/M + HCC

Cohort	Model	AUC (95% CI)	ACC	SEN	SPE	PPV	NPV	p^*^
Training cohort	Clinical model	0.636 (0.567–0.705)	0.619	0.497	0.729	0.646	0.604	**< 0.001**
	CT qualitative model	0.726 (0.660–0.790)	0.668	0.667	0.661	0.659	0.682	**0.046**
	CT quantitative model	0.749 (0.685–0.807)	0.690	0.706	0.671	0.673	0.709	0.156
	Combined model	0.784 (0.720–0.842)	0.722	0.697	0.739	0.728	0.723	**/**
External validation cohort	Clinical model	0.707 (0.620–0.793)	0.679	0.685	0.672	0.694	0.662	**0.005**
	CT qualitative model	0.724 (0.640–0.807)	0.650	0.658	0.642	0.667	0.632	**0.037**
	CT quantitative model	0.710 (0.623–0.796)	0.686	0.740	0.627	0.684	0.689	**0.024**
	Combined model	0.794 (0.718–0.870)	0.729	0.753	0.701	0.733	0.723	**/**

A *p* value less than 0.05 was considered statistically significant, presented in bold

Mean values of AUC, ACC, SEN, SPE, PPV, and NPV in the training cohort were used in bootstrap resampling (*n* = 1000)

*AUC* area under the curve, *CI* confidence interval, *ACC* accuracy, *SEN* sensitivity, *SPE* specificity, *PPV* positive predictive value, *NPV* negative predictive value, *V/M* VETC and/or MVI

^*^ DeLong test between the combinational model and the other three models in the original total cases

Among CT qualitative features, multivariable logistic regression demonstrated that non-smooth tumor margin (OR, 2.817; *p* = 0.001), the presence of internal arteries (OR, 2.556; *p* = 0.003), and intratumoral necrosis or severe ischemia (OR, 2.428; *p* = 0.004) were associated with V/M + HCC (Table S4). Using these three indicators, the CT qualitative model achieved training and validating AUCs of 0.726 and 0.724, respectively.

For CT quantitative features, larger tumor size (≥ 60 mm, OR, 3.146; *p* = 0.001), lower P-TLR (< 0.86, OR, 3.257; *p* < 0.001), and D-TLR (< 0.97, OR, 2.472; *p* = 0.020) were identified as independent predictors of V/M + HCC in the training cohort (Table S5). The CT quantitative model yielded training and external validation AUCs of 0.749 and 0.710, respectively.

**Fig. 5:**
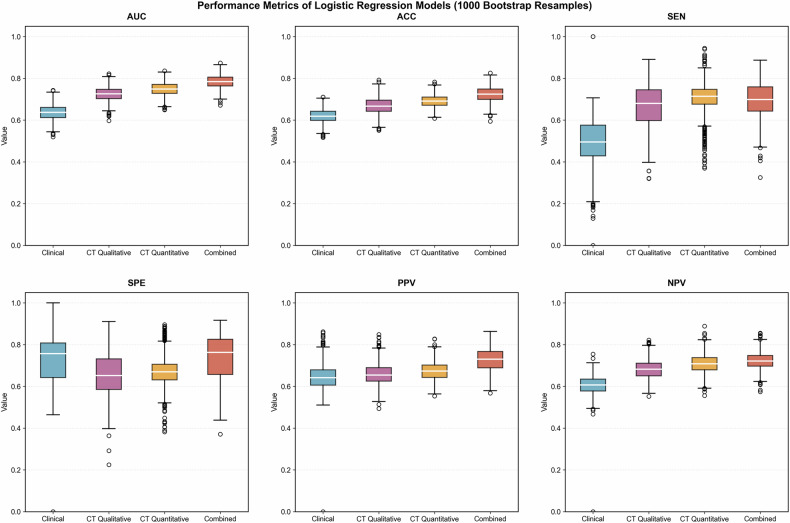
Internal validation of each V/M + HCC prediction model using bootstrap resampling (*n* = 1000) in the training cohort. Mean values across resamples were shown for AUC, ACC, SEN, SPE, PPV, and NPV. AUC, area under the curve; ACC, accuracy; SEN, sensitivity; SPE, specificity; PPV, positive predictive value; NPV, negative predictive value; V/M, VETC and/or MVI

Subsequently, all significant predictors from the clinical, CT qualitative, and CT quantitative models were entered into a multivariable logistic regression analysis to develop a final combined model, which identified four independent predictors: serum AFP ≥ 200 ng/mL, non-smooth tumor margin, internal arteries, and P-TLR < 0.86 (Table 4 and Fig. 5a). This combined model presented the best predictive performances, with a training mean AUC of 0.784 and a validating AUC of 0.794. DeLong tests showed significant differences in AUCs between the combined model and the other three models in both cohorts (*p* < 0.05), except for the CT quantitative model in the training cohort. A nomogram was constructed based on the combined model to facilitate individualized prediction of V/M + HCC risk (Fig. 5b). Calibration curves showed good agreement between predicted and observed outcomes (Fig. S1). Decision curve analysis demonstrated that the nomogram could provide improved clinical net benefit (Fig. S2).

**Table 4:** Training cohort uni- and multivariable logistic regression analyses for predicting V/M + HCC in the combined model

	Univariable			Multivariable				
	OR	95% CI	*p*	OR	95% CI	*p*	β	Score points
Age (< 50 years)	2.303	1.241–4.274	**0.008**			0.282		
AFP (≥ 200 ng/mL)	2.786	1.519–5.110	**0.001**	2.358	1.203–4.623	**0.012**	0.858	12
Tumor margin (non-smooth)	2.707	1.518–4.829	**0.001**	2.675	1.396–5.126	**0.003**	0.984	14
Internal arteries (present)	3.208	1.768–5.821	**< 0.001**	3.418	1.752-6.670	**< 0.001**	1.229	17
Intratumoral necrosis or severe ischemia (≥ 20%)	2.812	1.593–4.966	**< 0.001**			0.074		
Tumor size (≥ 60 mm)	3.193	1.733–5.885	**< 0.001**			0.090		
P-TLR (< 0.86)	3.874	2.136-7.026	**< 0.001**	4.208	2.163–8.186	**< 0.001**	1.437	20
D-TLR (< 0.97)	4.442	2.205–8.950	**< 0.001**			0.057		

A *p* value less than 0.05 was considered statistically significant, presented in bold

V/M+ score = 20 × P-TLR (< 0.86, 1; ≥ 0.86, 0) + 17 × internal arteries (present, 1; absent, 0) + 14 × non-smooth tumor margin (present, 1; absent, 0) + 12 × seurm AFP (≥ 200 ng/mL, 1; < 200 ng/mL, 0)

*AFP* alpha-fetoprotein, *OR* odds ratio, *CI* confidence interval, *P-TLR* tumor-to-liver density ratio in portal venous phase, *D-TLR* tumor-to-liver density ratio in delayed phase, *V/M* VETC and/or MVI

### Sensitivity analysis

A sensitivity analysis stratified by CT scanners revealed generally consistent performance across most scanners for each model. The combined model maintained the best discriminative ability, with AUCs ranging from 0.714 to 0.916 (Table S6).

The continuous-variable combined model, rebuilt with continuous features, identified age, non-smooth margin, internal arteries, and P-TLR as independent predictors, while serum AFP level was excluded as non-significant (Table S7). Compared to the categorical-variable combined model, both cohorts showed consistently lower discriminative performance, with slight declines in external validation AUC, ACC, SPE, PPV, and NPV (Table S8).

Another complementary sensitivity analysis using PSM was performed. After PSM, a total of 274 HCC patients were included, comprising 167 cases (82 V/M+ HCCs) and 107 cases (53 V/M+ HCCs) in the training and external validation cohorts, respectively. Baseline demographic and clinical characteristics were well balanced between the two cohorts post-PSM, with no statistically significant differences remaining (all *p* > 0.05; Table S9). All matched patients in the training cohort were applied to the four predictive models after PSM, demonstrating improved discriminatory performance (Table S10). Notably, the combined model maintained the highest discriminative ability (AUC = 0.782). Despite the superior overall performance of the combined model, no significant differences were observed between it and the CT quantitative model, similar to the performance pre-PSM.

### Patient’s follow-up and V/M+ score

By December 31, 2024, 325 of 347 (93.66%) patients had follow-up information. The overall 2-year recurrence rate was 52.31% (170/325 patients; training cohort: 50.52% [98/194 patients]; external validation cohort: 54.96% [72/131 patients]). According to the significant features from the optimal predictive model, the combined model, P-TLR < 0.86, was assigned 20 points, while internal arteries, non-smooth tumor margin, and serum AFP ≥ 200 ng/mL were assigned 17, 14, and 12 points, respectively (Table 3). Thus, the V/M+ score (Fig. S3) was formulated as: 20 × P-TLR (< 0.86, 1; ≥ 0.86, 0) + 17 × internal arteries (present, 1; absent, 0) + 14 × non-smooth tumor margin (present, 1; absent, 0) + 12 × serum AFP (≥ 200 ng/mL, 1; < 200 ng/mL, 0). The optimal threshold of the V/M+ score was 34 points, with scores ≥ 34 indicating high-risk patients for recurrence.

**Fig. 6:**
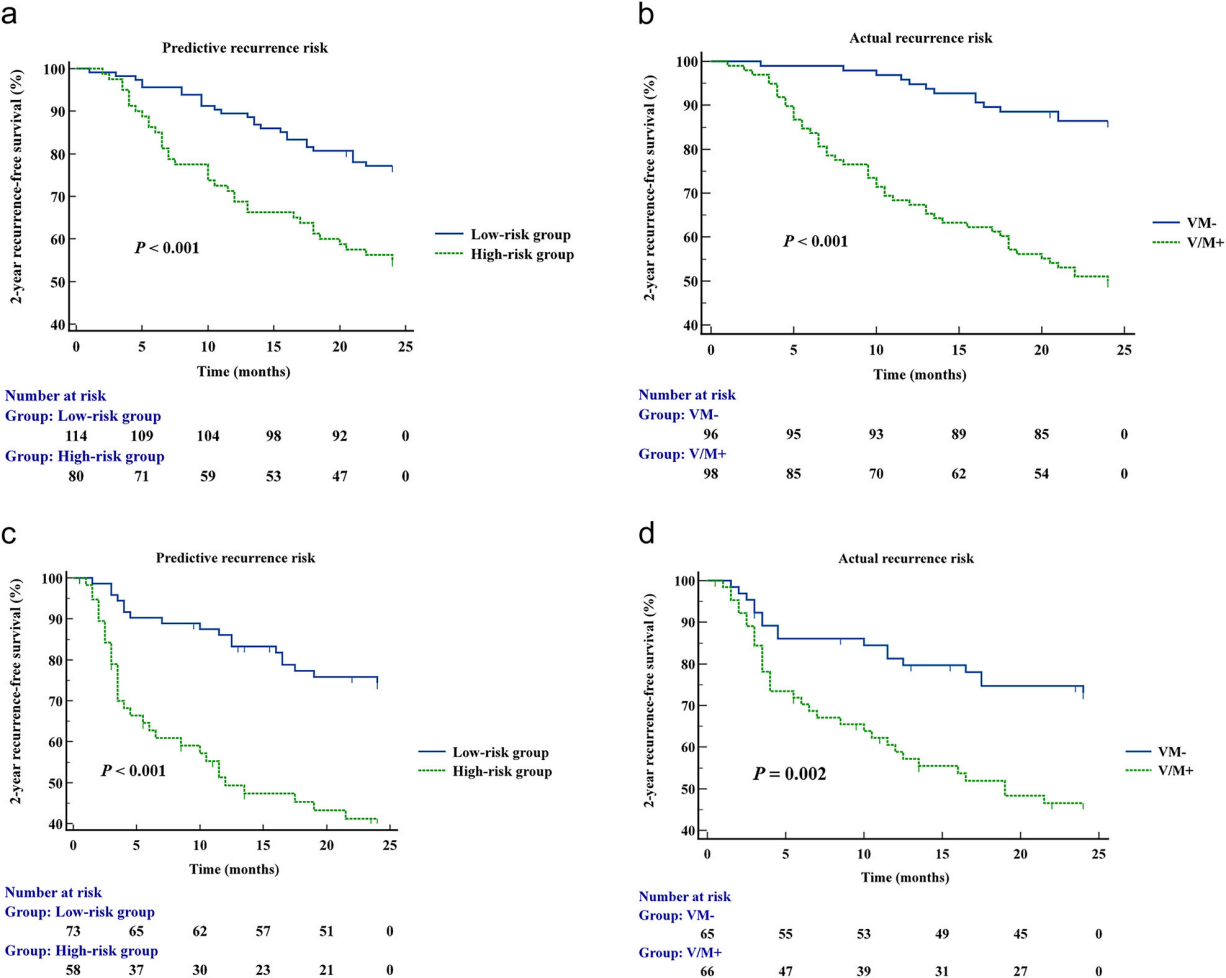
Kaplan–Meier curves of tumor RFS outcomes stratified by V/M+ score (**a**, **c**) and pathologically confirmed VM status (**b**, **d**), both in the training cohort (**a**, **b**) and the external validation cohort (**c**, **d**). Statistical comparison between survival curves was performed with the log-rank test. V/M, VETC and/or MVI

Kaplan–Meier survival analysis (Fig. 6) demonstrated that in both cohorts, the 2-year RFS were worse for patients with VM+ HCC and for those with the high-risk group based on V/M+ score (all *p* < 0.05). A consistent tendency was observed in the training cohort after PSM (*p* < 0.05, Fig. S4). In the multivariable Cox Proportional Hazards analysis that included the V/M+ score and variables excluded from final logistic models but significant in univariable analysis, Schoenfeld residuals detected no significant violations of the proportional hazards assumption (all *p* > 0.05). Tumor size ≥ 60 mm (hazard ratio [HR] = 2.204, *p* < 0.001) and a V/M+ score ≥ 34 (HR = 2.274, *p* < 0.001) were significantly associated with HCC recurrence (Table 5).

**Table 5:** Uni- and multivariable Cox proportional hazards analysis of parameters for predicting patients’ RFS

	Univariable HR (95% CI)	*p*	Multivariable HR (95% CI)	*p*
Age (< 50 years)	0.816 (0.543–1.225)	0.326		
Tumor size (≥ 60 mm)	2.710 (1.867–3.935)	**< 0.001**	2.204 (1.503–3.231)	**< 0.001**
Intratumoral necrosis or severe ischemia (≥ 20%)	1.373 (0.948–1.989)	0.094		
D-TLR (< 0.97)	0.469 (0.238–0.923)	**0.028**		
V/M+ score (≥ 34 points)	2.708 (1.852–3.960)	**< 0.001**	2.274 (1.539–3.360)	**< 0.001**

A *p* value less than 0.05 was considered statistically significant, presented in bold

*HR* hazard ratio, *CI* confidence interval, *D-TLR* tumor-to-liver density ratio in delayed phase, *V/M* VETC and/or MVI, *RFS* recurrence-free survival

## Discussion

In this study, by integrating serum AFP ≥ 200 ng/mL, non-smooth tumor margin, internal arteries, and P-TLR < 0.86, we established the best combined model (all AUCs > 0.77) for identifying V/M+ solitary HCC at the BCLC stage 0-A. The derived V/M+ score and tumor size ≥ 60 mm were found to be significantly associated with tumor recurrence. Notably, in the sensitivity analysis using PSM, both the combined model and V/M+ score maintained their effective power in the training cohort, with AUC remaining 0.782 and significant stratification of 2-year RFS, underscoring their stability in identifying V/M+ HCC and high-risk patients for recurrence, independent of inter-center variations in patient demographics or laboratory profiles.

V/M+ HCC is indicative of an aggressive tumor microenvironment and is strongly associated with poor oncological outcomes. Accurate preoperative identification of this phenotype is therefore critical for personalized therapies. Previous studies have highlighted the value of imaging biomarkers in detecting HCC V/M status [27, 28]. For instance, Zhu et al reported that serum AFP > 400 ng/mL, non-smooth tumor margin, and peritumoral arterial enhancement on MRI were independent predictors of V/M+ HCC, with AUCs > 0.79, though external validation was lacking [29]. Similarly, Yang et al emphasized that serum AFP > 400 ng/mL, intratumor vascularity, and contrast-enhancement patterns on DCE-MRI are key independent indicators of V/M+ HCC [30]. However, the widespread clinical application of MRI is limited by its high cost and patient contraindications, such as claustrophobia. Recently, Meng et al demonstrated that CT performed comparably to gadolinium ethoxybenzyl (EOB)-enhanced MRI in identifying VETC+ HCC, highlighting the potential of CT as an effective alternative imaging modality [31].

Among the CECT-derived features, non-smooth tumor margin emerged as a pivotal qualitative predictor, which represents aggressive histopathological characteristics such as extranodular growth, infiltrative borders, and poor encapsulation frequently associated with MVI+ and/or VETC+ phenotypes [18, 19, 29, 32, 33]. Aggressive HCC subtypes such as macrotrabecular-massive (MTM), scirrhous, and cytokeratin 19-positive variants often exhibited non-smooth tumor margins, which were associated with a higher occurrence of V/M+ status and tumor recurrence [34–37]. Thus, we hypothesized that a non-smooth tumor margin indicates a breach in the tumor capsule with outward invasion, corresponding to MVI+ regions, which could serve as a valuable surrogate for underlying pathological invasiveness and should be considered a radiological warning sign in clinical decision-making. Intratumoral arteries, visualized as tortuous, irregular vascular structures within the tumor on imaging, constituted a key imaging biomarker for V/M+ HCC in our study, aligning with previous research [38]. Unlike the orderly vascular architecture of normal liver tissue, intratumoral arteries reflect aberrant angiogenesis that is a hallmark of the VETC+ pattern [11]. According to Pan et al, these abnormal intratumoral arteries appeared more frequently in radiological images of VETC+ HCC compared to VETC- HCC, supporting our results [39]. It is speculated that the tortuous arteries visualized on CECT could directly indicate this pathological vascular remodeling. As demonstrated by Fang et al, these vessels not only serve for nourishment, but also provide a route for whole tumor clusters to enter the circulation, facilitating a unique and effective metastatic process [11].

Prior MRI-based studies have proven that quantitative measurements can serve as predictors for VETC+ HCC [23, 40]. However, the potential of CECT quantitative parameters for pre-treatment identification of V/M+ HCC remains underexplored. In this context, we identified P-TLR < 0.86 as a significant predictor of V/M+ solitary HCC in the BCLC stage 0-A. This finding aligned closely with the biological tendency of MVI to occur in portal vein branches. Notably, several imaging-based radiomics and deep learning studies have similarly confirmed that features derived from PVP outperformed those from other phases in predicting MVI [41–43]. In our study, P-TLR was measured in the region of the tumor demonstrating the greatest enhancement during AP. Compared to VM- HCC, V/M+ HCC in both datasets showed slightly reduced enhancement in the AP, even within the most enhancing tumor region. A marked reduction in enhancement was observed in the PVP, resulting in significantly lower P-TLR in V/M+ cases. This enhancement pattern may reflect altered tumor hemodynamics and perfusion, potentially due to mechanical compression of portal venules by the rapidly expanding mass, or underlying regions of ischemia—pathological features associated with V/M+ HCC. Interestingly, Wang et al observed that HCC with VETC+ patterns exhibited reduced microvascular density and increased tumor necrosis rates [44]. Similarly, Matsuda et al found that arterial vessel density (AVD) was significantly lower in VETC+ regions compared to VETC− regions [45]. The P-TLR, therefore, may uniquely capture the perfusion deficit associated with these aggressive vascular phenotypes.

Elevated serum AFP (> 200 ng/mL) was the only clinical predictor incorporated into the combined model, highlighting its crucial role in predicting VM status and patient outcomes. However, a sensitivity analysis that rebuilt the combined model with continuous candidate variables demonstrated slightly lower performance and did not retain AFP as an independent predictor, contrasting with our primary model and numerous prior studies [12, 25]. This indicates that the relationship between AFP and V/M+ status in HCC may be better captured by a clinically established threshold effect rather than a linear association, with dichotomization at a relevant cut-off offering a more effective model for clinical decision-making. Tumor size ≥ 60 mm was recognized as an independent risk factor for patients’ RFS in this study, similar to most malignant tumors. Larger tumor volume often suggests advanced disease progression with a higher risk of postoperative recurrence and metastasis, aligning with existing HCC staging systems [3, 17].

Our developed combined model and V/M+ score are crucial for optimizing treatment strategies, delineating surgical margins, and planning comprehensive follow-up. For instance, preoperative identification of V/M+ HCC enables precise selection of liver-transplant candidates. Patients with V/M+ tumors should be excluded or down-staged before transplantation, as they have a high risk of early recurrence and transplantation failure [46]. Likewise, in candidates scheduled for ablation, a V/M+ HCC diagnosis can prompt an intraoperative wider ablative margin and postoperative combination therapy to reduce the possibility of local recurrence and micrometastases [47]. Moreover, V/M+ score-based stratification enables clinicians to tailor surveillance and therapy strategies. An intensive surveillance regimen, and even aggressive or expensive preventive and adjuvant therapies like programmed cell death protein-1 inhibitors and multitargeted tyrosine kinase inhibitors, can be considered to prolong patients' overall survival [48, 49]. While low-risk patients may receive lighter surveillance and more selective use of expensive therapies, thereby reducing both adverse events and financial burden.

This study has several limitations. First, the retrospective design may introduce selection bias. Second, CT quantitative measurements were confined to the most enhanced region of the tumor during AP, rather than encompassing the entire tumor, potentially overlooking regional heterogeneity and additional features indicative of vascular invasion. Employing and comparing diverse measurement strategies or artificial intelligence-based feature-extraction approaches is promising for optimizing the identification of V/M+ HCC. Third, categorizing continuous predictors like serum AFP to make clinical interpretation easier inevitably reduces statistical information. A complementary sensitivity analysis using continuous forms of these variables produced a model with inferior performance, highlighting the clinical relevance of the categorical variables-based model’s structure. Fourth, the study was restricted to pathologically confirmed, solitary early-stage HCC treated with surgical resection, limiting the generalizability to multifocal, recurrent, or metastatic cases. A broader patient population (e.g., non-surgical patients) must be investigated to verify the combined model’s prognostic value and facilitate broader clinical translation. Fifth, the biological mechanisms linking CECT-derived imaging features to V/M+ HCC pathology remain insufficiently elucidated. Future large-scale, multicenter, multi-modality fusion (e.g., MRI) and prospective investigations combining radiomics, pathology, and genomics using systematic and objective variable selection approaches, and more robust statistical methods are needed to validate and further elucidate these imaging indicators and derive the most parsimonious and robust final model.

In conclusion, our study demonstrated that a combination of CECT qualitative and quantitative features, along with serum AFP ≥ 200 ng/mL, can effectively identify V/M+ status in solitary BCLC stage 0-A HCC and patients’ recurrence risk before treatment. This comprehensive assessment approach facilitates non-invasive, preoperative prediction of HCC's distinct vascular invasion characteristics and prognosis, especially in surgical candidates with early-stage HCC, offering a reliable imaging foundation to support precision diagnosis and facilitate personalized treatment planning in clinical practice.

## Supplementary information

Supplementary information

## Data Availability

The datasets used for analyses during this study are available from the corresponding author upon reasonable request.

## Abbreviations

AAR: Aspartate aminotransferase-to-alanine aminotransferase ratio
ACC: Accuracy
AFP: Alpha-fetoprotein
ALT: Alanine aminotransferase
AP: Arterial phase
AST: Aspartate aminotransferase
AUC: Area under the curve
BCLC: Barcelona Clinic Liver Cancer
CECT: Contrast-enhanced computed tomography
DP: Delayed phase
EMT: Epithelial-mesenchymal transition
ES: Edmondson-Steiner
GAR: γ-glutamyl transpeptidase-to-aspartate aminotransferase ratio
GGT: γ-glutamyl transpeptidase
GLR: γ-glutamyl transpeptidase-to-lymphocyte ratio
HCC: Hepatocellular carcinoma
ICC: Intraclass correlation coefficient
LI-RADS: Liver imaging reporting and data system
MTM: Macrotrabecular-massive
MVI: Microvascular invasion
NLR: Neutrophil-to-lymphocyte ratio
NPV: Negative predictive value
PLR: Platelet-to-lymphocyte ratio
PPV: Positive predictive value
PSM: Propensity score matching
P-TLR: Density ratio of tumor-to-liver in portal venous phase
PVP: Portal venous phase
PWR: Platelet-to-white blood cell ratio
RBC: Red blood cell
RFS: Recurrence-free survival
ROC: Receiver operating characteristic
SEN: Sensitivity
SPE: Specificity
STAR: Standardized tumor-to-aorta density ratio
TAR: Tumor-to-aorta density ratio
TLR: Tumor-to-liver density ratio
V/M+: MVI and/or VETC-positive
VETC: Vessels that encapsulate tumor clusters
WBC: White blood cell
