# Supplementary material for: Preoperative contrast-enhanced CT prediction of distinct vascular patterns in solitary early-stage hepatocellular carcinoma and its prognostic value
Source: Insights Imaging. 2026 Feb 16;17:49. doi: 10.1186/s13244-026-02224-5 (PMC12909690; doi:10.1186/s13244-026-02224-5)

**Preoperative Contrast-Enhanced CT Prediction of Distinct Vascular Patterns in Solitary Early-stage Hepatocellular Carcinoma and Its Prognostic Value**

**ELECTRONIC SUPPLEMENTARY MATERIAL**

**Appendix S1**

**Contrast-enhanced CT (CECT) imaging parameters**

Preoperative images were acquired using CECT scanners, including the GE LightSpeed 16, Toshiba Aquilion One 16, Toshiba Aquilion One 320, Philips Brilliance 64, Philips iCT 256, GE Revolution CT, and GE Discovery 64. All scanners used standardized scanning protocols with parameters including a tube voltage of 120 kVp, a tube current of 200 mAs, and a reconstruction slice thickness of 5 mm. After unenhanced CT images were taken, each patient was injected intravenously with a nonionic iodinated contrast agent (iodipamide, 370 mg I/mL, Bracco) through the antecubital vein, delivered by mechanical power injectors based on their weight (2.0 mL/kg body weight, with a maximal dose of 180ml), and then a 20-mL saline flush was administered. Finally, three-phase CECT imaging was acquired, consisting of the arterial phase (AP, 30-40s), portal venous phase (PVP, 60-90s), and delayed phase (DP,120-250s).

**Appendix S2**

**Methods of CECT image features measurements**

Two board-certified radiologists (Wanli Zhang and Ruimeng Yang, with 7 and 20 years of experience in liver CECT, respectively) independently reviewed all CECT images. Before the assessment, they participated in an extensive, day-long session that combined lectures and practical exercises, detailing the qualitative features and quantitative measurements.

For the quantitative measurements, the density in each region of interest (ROI) from each CECT phase was measured using a DICOM viewer. A circular or elliptical ROI [median (IQR), with a median area of 26.07 mm^2^ (IQR, 15.22, 46.94), was placed in the tumor region with the most significant enhancement during the AP. The identical ROI was subsequently utilized for the PVP and DP, producing density measurements for each lesion throughout the three phases of CECT. Later, extra ROIs were set within the liver parenchyma and aorta at the very same level of the three enhanced CT phases, with careful avoidance of vascular structures, bile ducts, artifacts, and localized lesions, for density measurement of the liver and aorta. Minor adjustments were allowed on all images to tailor the ROIs. Each reviewer independently placed all ROIs three times, and the final measurement for each ROI was determined by calculating the mean of these obtained values.

Ultimately, the density of lesion, liver parenchyma and aorta would achieve a total of 9 quantitative features, as described below:

1. the tumor-to-liver density ratio (TLR) in AP: A-TLR = $\frac{\text{Tumor}\text{ (}\text{Hu}\text{)}}{\text{Liver}\text{ (}\text{Hu}\text{)}}$
2. the tumor-to-liver density ratio (TLR) in PVP: P-TLR = $\frac{\text{Tumor}\text{ (}\text{Hu}\text{)}}{\text{Liver}\text{ (}\text{Hu}\text{)}}$
3. the tumor-to-liver density ratio (TLR) in DP: D-TLR = $\frac{\text{Tumor}\text{ (}\text{Hu}\text{)}}{\text{Liver}\text{ (}\text{Hu}\text{)}}$
4. the tumor-to-aorta density ratio (TAR) in AP: A-TAR = $\frac{\text{Tumor}\text{ (}\text{Hu}\text{)}}{\text{Aorta}\text{ (}\text{Hu}\text{)}}$
5. the tumor-to-aorta density ratio (TAR) in PVP: P-TAR = $\frac{\text{Tumor}\text{ (}\text{Hu}\text{)}}{\text{Aorta}\text{ (}\text{Hu}\text{)}}$
6. the tumor-to-aorta density ratio (TAR) in DP: D-TAR = $\frac{\text{Tumor}\text{ (}\text{Hu}\text{)}}{\text{Aorta}\text{ (}\text{Hu}\text{)}}$
7. the standardized tumor-to-aorta density ratio (STAR) in AP: A-STAR = $\frac{\text{(}\text{Tumor}\text{−}\text{Liver}\text{) (}\text{Hu}\text{)}}{\text{Aorta}\text{ (}\text{Hu}\text{)}}$
8. the standardized tumor-to-aorta density ratio (STAR) in PVP: P-STAR = $\frac{\text{(}\text{Tumor}\text{−}\text{Liver}\text{) (}\text{Hu}\text{)}}{\text{Aorta}\text{ (}\text{Hu}\text{)}}$
9. the standardized tumor-to-aorta density ratio (STAR) in DP: D-STAR = $\frac{\text{(}\text{Tumor}\text{−}\text{Liver}\text{) (}\text{Hu}\text{)}}{\text{Aorta}\text{ (}\text{Hu}\text{)}}$

**Appendix S3**

**Propensity Score Matching for subgroup analysis**

To mitigate potential confounding and selection bias within the multicenter study, propensity score matching (PSM) was performed to harmonize data from the two medical centers. Specifically, a 1:2 (training cohort vs. external validtion cohort) nearest-neighbor matching without replacement was implemented to ensure precise and independent matching. During this process, a caliper value of 0.2 was applied to limit the differences in propensity scores between matched pairs, thereby constructing a well-balanced propensity score-matched subgroup cohort. Subsequently, the four predictive models were applied to this PSM-adjusted subgroup in the training cohort to rigorously evaluate their performance within this specific population.

**Table S1. Contrast-enhanced CT qualitative and quantitative imaging features**

| **Features** | **Definition** |
| --- | --- |
| **Qualitative features** |  |
| Tumor margin | The tumor contour might be oval or circular with smooth margin, or it could be irregular, with extranodular extension, invasive margin, or multinodular appearance (non-smooth margin). |
| Capsule | A smooth, homogeneous, and sharply defined margin that encompasses the entire lesion or a large portion of the lesion on CT. |
| Blood products in mass | Amorphous or geographic hyperattenuation relative to liver in acute and subacute phase, iso or hypoattenuation in chronic phase of unenhanced CT. |
| Fat in mass | On unenhanced CT, mass or part of mass has attenuation < (-10) HU or mass has attenuation less than liver if liver is fatty. |
| APHE | Enhancement in AP more than liver, resulting in brightness higher than liver. |
| Rim APHE | Spatially defined subtype of APHE in which AP enhancement is most pronounced in observation periphery. |
| Corona enhancement | Periobservational enhancement in AP or PVP, which is contiguous with and surrounds all or part of the observation. |
| Fade | Reduction in enhancement relative to liver from hyperehancement during the AP or PVP on CT to isoenhancement or minimal hyperenhancement in all subsequent phases. |
| Peripheral washout | Reduction in enhancement from hyper- or iso-enhancement during the AP to hyporenhancement during the PVP or DP, which is mainly in observation periphery. |
| Mosaic architecture | Presence of any combination of internal nodules, compartments, or septations, within a solid or mostly solid mass. |
| Nodule-in-nodule | Presence of a smaller inner nodule within a larger outer nodule. |
| Intratumoral necrosis or severe ischemia | A central area with low or iso-density on the unenhanced phase exhibits slight or no enhancement on contrast-enhanced phases, affecting at least 20% of the tumor's area. |
| Targetoid sign | Target-like morphology on CT. The center and periphery of a mass have different imaging characteristics. |
| Hypodense halo | A hypodense shadow surrounding the tumor in the PVP or DP. |
| Internal arteries | Hyperattenuation of arteries within lesions in the AP. |
| TTPVI | The presence of “internal arteries” and absence of “hypodense halos”. |
| Perfusion alteration | Nonmasslike change in blood supply to an area of the liver. |
| **Quantitative features** |  |
| TLR | Tumor-to-liver density ratio (tumor/liver) in AP, PVP and DP, respectively. |
| TAR | Tumor-to-aorta density ratio (tumor/aorta) in AP, PVP and DP, respectively. |
| STAR | Standardized tumor-to-aorta density ratio ((tumor-liver)/aorta) in AP, PVP and DP, respectively. |

AP, arterial phase; APHE, arterial phase hyperenhancement; PVP, portal venous phase; DP, delayed phase; TTPVI, two-trait predictor of venous invasion; TLR, tumor-to-liver density ratio; TAR, tumor-to-aorta density ratio; STAR, standardized tumor-to-aorta density ratio.

**Table S2. Inter-observer agreement of CECT features**

|  | Cohen's kappa coefficient / ICC (95% CI) |
| --- | --- |
| **Qualitative imaging features** |  |
| Tumor margin | 0.728 (0.591-0.865) |
| Capsule | 0.714 (0.559-0.869) |
| Blood products in mass | 0.779 (0.538-1.021) |
| Fat in mass | 1 (1.000-1.000) |
| APHE | 0.745 (0.623-0.867) |
| Rim APHE | 0.715 (0.592-0.838) |
| Corona enhancement | 0.765 (0.687-0.843) |
| Fade | 0.934 (0.807-1.061) |
| Peripheral washout | 0.687 (0.531-0.843) |
| Mosaic architecture | 0.681 (0.522-0.840) |
| Nodule-in-nodule | 0.763 (0.640-0.886) |
| Intratumoral necrosis or severe ischemia (≥ 20%) | 0.774 (0.627-0.921) |
| Intratumoral necrosis or severe ischemia (≥ 50%) | 0.783 (0.719-0.847) |
| Targetoid sign | 0.774 (0.547-1.001) |
| Internal arteries | 0.73 (0.536-0.924) |
| Hypodense halo | 0.801 (0.710-0.892) |
| TTPVI | 0.759 (0.644-0.874) |
| Perfusion alteration | 0.844 (0.770-0.916) |
| **Quantitative imaging features** |  |
| Tumor size (mm) | 0.972 (0.960-0.981) |
| A-TLR | 0.729 (0.607-0.818) |
| P-TLR | 0.872 (0.807-0.916) |
| D-TLR | 0.780 (0.676-0.853) |
| A-TAR | 0.906 (0.856-0.939) |
| P-TAR | 0.844 (0.767-0.897) |
| D-TAR | 0.794 (0.697-0.863) |
| A-STAR | 0.740 (0.622-0.826) |
| P-STAR | 0.849 (0.773-0.901) |
| D-STAR | 0.753 (0.640-0.835) |

ICC, intraclass correlation; APHE, arterial phase hyperenhancement; TTPVI, two-trait predictor of venous invasion; A, arterial phase; P, portal venous phase; D, delayed phase; TLR, tumor-to-liver density ratio; TAR, tumor-to-aorta density ratio; STAR, standardized tumor-to-aorta density ratio.

**Table S3. Uni- and multivariable logistic regression analyses for predicting V/M+ HCC in the clinical model within the training cohort**

|  | Univariable | | | Multivariable | | |
| --- | --- | --- | --- | --- | --- | --- |
|  | OR | 95% CI | *P* | OR | 95% CI | *P* |
| Age ( < 50 years) | 2.303 | 1.241-4.274 | **0.008** | 1.943 | 1.025-3.683 | **0.042** |
| Hepatic virus infection (present) | 2.200 | 1.059-4.571 | **0.035** |  |  |  |
| AFP ( ≥ 200 ng/mL) | 2.786 | 1.519-5.110 | **0.001** | 2.476 | 1.331-4.604 | **0.004** |

A *P* value less than 0.05 was considered statistically significant.

AFP, alpha-fetoprotein; OR, Odds Ratio; CI, confidence interval; V/M, vessels that encapsulate tumor clusters (VETC) and/or microvascular invasion (MVI).

**Table S4. Uni- and multivariable logistic regression analyses for predicting V/M+ HCC in the CT qualitative model within the training cohort**

|  | Univariable | | | Multivariable | | |
| --- | --- | --- | --- | --- | --- | --- |
|  | OR | 95% CI | *P* | OR | 95% CI | *P* |
| Tumor margin (non-smooth) | 2.707 | 1.518-4.829 | **0.001** | 2.817 | 1.520-5.220 | **0.001** |
| Internal arteries (present) | 3.208 | 1.768-5.821 | **< 0.001** | 2.556 | 1.365-4.788 | **0.003** |
| TTPVI (positive) | 2.645 | 1.499-4.667 | **0.001** |  |  |  |
| Intratumoral necrosis or severe ischemia (≥ 50%) | 2.777 | 1.371-5.625 | **0.005** |  |  |  |
| Intratumoral necrosis or severe ischemia (≥ 20%) | 2.812 | 1.593-4.966 | **< 0.001** | 2.428 | 1.319-4.469 | **0.004** |
| Corona enhancement (present) | 4.196 | 1.135-15.514 | **0.032** |  |  |  |

A *P* value less than 0.05 was considered statistically significant.

AFP, alpha-fetoprotein; OR, Odds Ratio; CI, confidence interval; TTPVI, two-trait predictor of venous invasion; V/M, vessels that encapsulate tumor clusters (VETC) and/or microvascular invasion (MVI).

**Table S5. Uni- and multivariable logistic regression analyses for predicting V/M+ HCC in the CT quantitative model within the training cohort**

|  | **Univariable** | | | **Multivariable** | | |
| --- | --- | --- | --- | --- | --- | --- |
|  | OR | 95% CI | *P* | OR | 95% CI | *P* |
| Tumor size (≥ 60 mm) | 3.193 | 1.733-5.885 | **< 0.001** | 3.146 | 1.619-6.113 | **0.001** |
| A-TAR(< 0.25) | 1.983 | 1.133-3.473 | **0.017** |  |  |  |
| A-STAR (< 0.05) | 2.247 | 1.278-3.952 | **0.005** |  |  |  |
| P-TLR (< 0.86) | 3.874 | 2.136-7.026 | **< 0.001** | 3.257 | 1.681-6.309 | **< 0.001** |
| P-TAR (< 0.65) | 4.089 | 2.155-7.759 | **< 0.001** |  |  |  |
| P-STAR [< (-0.11)] | 3.362 | 1.870-6.042 | **< 0.001** |  |  |  |
| D-TLR (< 0.97) | 4.442 | 2.205-8.950 | **< 0.001** | 2.472 | 1.152-5.302 | **0.020** |
| D-TAR (< 0.74) | 1.837 | 1.058-3.190 | **0.031** |  |  |  |
| D-STAR [< (-0.02)] | 4.145 | 2.014-8.529 | **< 0.001** |  |  |  |

A *P* value less than 0.05 was considered statistically significant.

OR, Odds Ratio; CI, confidence interval; TAR, tumor-to-aorta density ratio; TLR, tumor-to-liver density ratio; STAR, standardized tumor-to-aorta density ratio; A, arterial phase; P, portal venous phase; D, delayed phase; V/M, vessels that encapsulate tumor clusters (VETC) and/or microvascular invasion (MVI).

**Table S6. Performances of the four models for predicting V/M+ HCC stratified by CT scanner**

| **Cohort** | **CT scanner** | **Model** | **AUC (95% CI)** | **ACC** | **SEN** | **SPE** | **PPV** | **NPV** |
| --- | --- | --- | --- | --- | --- | --- | --- | --- |
| **Training cohort** | **All CT scanners (n = 207, 101 V/M+ HCC)** | Clinical model | 0.634 (0.564-0.700) | 0.599 | 0.574 | 0.623 | 0.592 | 0.606 |
|  |  | CT qualitative model | 0.717 (0.650-0.777) | 0.657 | 0.762 | 0.557 | 0.621 | 0.711 |
|  |  | CT quantitative model | 0.741 (0.676-0.800) | 0.686 | 0.703 | 0.670 | 0.670 | 0.703 |
|  |  | Combined model | 0.778 (0.715-0.832) | 0.729 | 0.653 | 0.802 | 0.759 | 0.708 |
|  | **Philips iCT 256 (n = 100, 53 V/M+ HCC)** | Clinical model | 0.674 (0.569-0.779) | 0.640 | 0.623 | 0.660 | 0.673 | 0.608 |
|  |  | CT qualitative model | 0.715 (0.614-0.815) | 0.660 | 0.755 | 0.553 | 0.656 | 0.667 |
|  |  | CT quantitative model | 0.752 (0.657-0.847) | 0.700 | 0.698 | 0.702 | 0.725 | 0.673 |
|  |  | Combined model | 0.792 (0.704-0.880) | 0.680 | 0.774 | 0.574 | 0.672 | 0.692 |
|  | **Revolution CT (n = 34, 13 V/M+ HCC)** | Clinical model | 0.685 (0.490-0.880) | 0.676 | 0.615 | 0.714 | 0.571 | 0.750 |
|  |  | CT qualitative model | 0.804 (0.658-0.950) | 0.706 | 0.692 | 0.714 | 0.600 | 0.789 |
|  |  | CT quantitative model | 0.795 (0.635-0.955) | 0.735 | 0.692 | 0.762 | 0.643 | 0.800 |
|  |  | Combined model | 0.916 (0.826-1.00) | 0.824 | 0.692 | 0.905 | 0.818 | 0.826 |
|  | **GE Discovery 64 (n = 46, 22 V/M+ HCC)** | Clinical model | 0.665 (0.508-0.822) | 0.630 | 0.364 | 0.875 | 0.727 | 0.600 |
|  |  | CT qualitative model | 0.694 (0.541-0.847) | 0.609 | 0.455 | 0.750 | 0.625 | 0.600 |
|  |  | CT quantitative model | 0.708 (0.559-0.858) | 0.652 | 0.409 | 0.875 | 0.750 | 0.618 |
|  |  | Combined model | 0.714 (0.566-0.862) | 0.565 | 0.500 | 0.625 | 0.550 | 0.577 |
| **External validation cohort** | **All CT scanners (n = 140, 73 V/M+ HCC)** | Clinical model | 0.707 (0.620-0.793) | 0.679 | 0.685 | 0.672 | 0.694 | 0.662 |
|  |  | CT qualitative model | 0.724 (0.640-0.807) | 0.650 | 0.658 | 0.642 | 0.667 | 0.632 |
|  |  | CT quantitative model | 0.710 (0.623-0.796) | 0.686 | 0.740 | 0.627 | 0.684 | 0.689 |
|  |  | Combined model | 0.794 (0.718-0.870) | 0.729 | 0.753 | 0.701 | 0.733 | 0.723 |
|  | **Toshiba Aquilion one 320 (n = 121, 63 V/M+ HCC)** | Clinical model | 0.701 (0.607-0.795) | 0.669 | 0.540 | 0.810 | 0.756 | 0.618 |
|  |  | CT qualitative model | 0.762 (0.676-0.848) | 0.711 | 0.746 | 0.672 | 0.712 | 0.709 |
|  |  | CT quantitative model | 0.716 (0.623-0.808) | 0.694 | 0.746 | 0.638 | 0.691 | 0.698 |
|  |  | Combined model | 0.813 (0.733-0.892) | 0.744 | 0.746 | 0.741 | 0.758 | 0.729 |

*^*^* DeLong test between combinational model and other three models.

AUC, area under the curve; CI, confidence interval; ACC, accuracy; SEN, sensitivity; SPE, specificity; PPV, positive predictive value; NPV, negative predictive value; V/M, vessels that encapsulate tumor clusters (VETC) and/or microvascular invasion (MVI).

**Table S7. Training cohort uni- and multivariable logistic regression analyses for predicting V/M+ HCC with continuous variables in the combined model**

|  | **Univariable** | | | **Multivariable** | | |
| --- | --- | --- | --- | --- | --- | --- |
|  | OR | 95% CI | *P* | OR | 95% CI | *P* |
| Age | 0.963 | 0.940-0.987 | **0.003** | 0.037 | 0.939-0.992 | **0.011** |
| AFP | 1.000 | 1.000-1.001 | **0.013** |  |  |  |
| Tumor margin (non-smooth) | 2.707 | 1.518-4.829 | **0.001** | 2.564 | 1.364-4.817 | **0.003** |
| Internal arteries (present) | 3.208 | 1.768-5.821 | **< 0.001** | 3.435 | 1.791-6.587 | **< 0.001** |
| Intratumoral necrosis or severe ischemia (≥ 20%) | 2.570 | 1.463-4.517 | **< 0.001** |  |  |  |
| Tumor size | 1.014 | 1.005-1.023 | **0.002** |  |  |  |
| P-TLR | 0.023 | 0.003-0.176 | **< 0.001** | 0.037 | 0.004-0.310 | **0.002** |
| D-TLR | 0.018 | 0.002-0.203 | **0.001** |  |  |  |

A *P* value less than 0.05 was considered statistically significant.

The detailed OR of serum AFP level in the univariable logistic regresion was 1.000302 (95% CI:1.000062-1.000541)，*P* = 0.013.

AFP, alpha-fetoprotein; OR, Odds Ratio; CI, confidence interval; P-TLR, tumor-to-liver density ratio in portal venous phase; D-TLR, tumor-to-liver density ratio in delayed phase; V/M, vessels that encapsulate tumor clusters (VETC) and/or microvascular invasion (MVI).

**Table S8. Performances of the combined models for predicting V/M+ HCC with different variables types**

| **Cohort** | **Variable types** | **AUC (95% CI)** | **ACC** | **SEN** | **SPE** | **PPV** | **NPV** |
| --- | --- | --- | --- | --- | --- | --- | --- |
| Training cohort | Categorical variables | 0.778 (0.715-0.832) | 0.729 | 0.653 | 0.802 | 0.759 | 0.708 |
|  | Categorical variables with 1000 bootstrap resamples | 0.784 (0.720-0.842) | 0.722 | 0.697 | 0.739 | 0.728 | 0.723 |
|  | Continuous variables | 0.763 (0.699-0.828) | 0.691 | 0.673 | 0.708 | 0.687 | 0.694 |
| External validation cohort | Categorical variables | 0.794 (0.718-0.870) | 0.729 | 0.753 | 0.701 | 0.733 | 0.723 |
|  | Continuous variables | 0.762 (0.683-0.842) | 0.686 | 0.726 | 0.642 | 0.688 | 0.683 |

AUC, area under the curve; CI, confidence interval; ACC, accuracy; SEN, sensitivity; SPE, specificity; PPV, positive predictive value; NPV, negative predictive value; V/M, vessels that encapsulate tumor clusters (VETC) and/or microvascular invasion (MVI).

**Table S9. Demographic and clinical characteristics of HCC patients pre- and post-PSM**

|  | Pre-PSM | | | Post-PSM | | |
| --- | --- | --- | --- | --- | --- | --- |
|  | Training cohort  (n = 207) | External validation cohort (n = 140) | *P* | Training cohort  (n = 167) | External validation cohort (n = 107) | *P* |
| WBC (10^9^/L) ^b^ | 6.10 (4.99, 7.47) | 6.37 (5.29, 7.86) | 0.108 | 6.18 (5.08, 7.54) | 6.24 (5.08, 7.54) | 0.984 |
| RBC (10^12^/L) ^b^ | 4.57 (4.13, 5.03) | 4.52 (4.12, 5.05) | 0.816 | 4.61 (4.12, 5.03) | 4.49 (4.22, 4.99) | 0.752 |
| Hemoglobin (g/L) ^b^ | 136.00 (123.50, 149.00) | 137.50 (120.75, 151.00) | 0.990 | 136.00 (122.50, 150.00) | 138.00 (124.50, 150.50) | 0.757 |
| PLT (10^9^/L) ^b^ | 197.00 (158.00, 241.00) | 191.50 (149.75, 252.50) | 0.927 | 197.00 (160.50, 243.00) | 190.00 (150.00, 248.50) | 0.891 |
| Neutrophil (10^9^/L) ^b^ | 3.58 (2.81, 4.78) | 3.79 (2.93, 4.93) | 0.384 | 3.66 (2.95, 4.78) | 3.76 (2.79, 4.70) | 0.798 |
| Lymphocyte (10^9^/L) ^b^ | 1.57 (1.23, 1.93) | 1.62 (1.18, 2.06) | 0.610 | 1.58 (1.29, 1.98) | 1.62 (1.25, 2.04) | 0.879 |
| PLT/WBC ^b^ | 30.96 (24.17, 39.26) | 30.54 (23.37, 37.61) | 0.418 | 30.63 (24.01, 38.60) | 31.90 (24.09, 37.39) | 0.707 |
| PLR ^b^ | 122.15 (96.99, 160.51) | 116.74 (93.74, 170.77) | 0.716 | 117.26 (94.84, 155.95) | 115.53 (96.56, 154.36) | 0.975 |
| NLR ^b^ | 2.28 (1.73, 3.16) | 2.36 (1.68, 3.28) | 0.866 | 2.28 (1.73, 3.19) | 2.34 (1.71, 3.10) | 0.999 |
| GLR ^b^ | 34.83 (19.62, 64.22) | 41.22 (21.89, 89.69) | **0.028** | 36.84 (20.84, 70.25) | 36.28 (20.62, 86.44) | 0.525 |
| ALT ^b^ | 29.00 (20.00, 43.50) | 31.50 (21.00, 45.25) | 0.673 | 31.00 (21.50, 45.00) | 31.00 (20.50, 46.50) | 0.797 |
| AST ^b^ | 31.00 (24.00, 42.50) | 35.50 (27.75, 53.00) | **0.004** | 32.00 (25.50, 44.50) | 33.00 (27.00, 48.00) | 0.392 |
| GGT ^b^ | 51.00 (30.00, 84.00) | 67.00 (36.00, 140.00) | **0.007** | 57.00 (31.50, 93.00) | 60.00 (33.00, 114.50) | 0.500 |
| AST/ALT ^b^ | 1.07 (0.84, 1.36) | 1.13 (0.96, 1.53) | **0.006** | 1.07 (0.83, 1.37) | 1.09 (0.96, 1.47) | 0.125 |
| GGT/AST ^b^ | 1.60 (1.02, 2.36) | 1.73 (1.04, 3.04) | 0.379 | 1.62 (1.07, 2.62) | 1.67 (1.03, 2.85) | 0.994 |
| Albumin (g/L) ^b^ | 40.40 (37.95, 42.55) | 37.80 (34.90, 41.15) | **< 0.001** | 39.80 (37.65, 42.00) | 39.00 (35.90, 41.70) | 0.065 |
| Gender ^*^ |  |  | 0.860 |  |  | 0.990 |
| male | 173 (83.57) | 118 (84.29) |  | 139 (83.23) | 89 (83.18) |  |
| female | 34 (16.43) | 22 (15.71) |  | 28 (16.77) | 18 (16.82) |  |
| Age (year) ^*^ |  |  | 0.414 |  |  | 0.798 |
| < 50 | 60 (28.99) | 35 (25.00) |  | 46 (27.54) | 31 (28.97) |  |
| ≥ 50 | 147 (71.01) | 105 (75.00) |  | 121 (72.46) | 76 (71.03) |  |
| Hepatic virus infection^*^ |  |  | 0.950 |  |  | 0.595 |
| absent | 39 (18.84) | 26 (18.57) |  | 33 (19.76) | 24 (22.43) |  |
| present | 168 (81.16) | 114 (81.43) |  | 134 (80.24) | 83 (77.57) |  |
| AFP (ng/mL)^*^ |  |  | 0.292 |  |  | 0.808 |
| < 200 | 140 (67.63) | 87 (62.14) |  | 110 (65.87) | 72 (67.29) |  |
| ≥ 200 | 67 (32.37) | 53 (37.86) |  | 57 (34.13) | 35 (32.71) |  |

^*^Chi-square test. Data are number of tumors, with percentages in parentheses.

^b^ Mann-Whitney U test. Data are median (interquartile range, IQR).

A *P* value less than 0.05 was considered statistically significant, presented in **bold**.

AFP, alpha-fetoprotein; WBC, white blood cell; RBC, red blood cell; PLT, platelet; ALT, alanine aminotransferase; AST, aspartate aminotransferase; GGT, γ-glutamyl transpeptidase; PLR, PLT-to-Lymphocyte Ratio; NLR, Neutrophil-to-Lymphocyte Ratio; GLR, GGT-to-Lymphocyte Ratio; PSM, propensity score matching.

**Table S10. Performances of the four models for predicting V/M+ HCC after PSM in the training cohort**

| Model | AUC (95% CI) | ACC | SEN | SPE | PPV | NPV | *P^*^* |
| --- | --- | --- | --- | --- | --- | --- | --- |
| Clinical model | 0.630 (0.544-0.715) | 0.623 | 0.463 | 0.776 | 0.667 | 0.600 | **< 0.001** |
| CT qualitative model | 0.694 (0.615-0.773) | 0.635 | 0.768 | 0.506 | 0.600 | 0.694 | **0.016** |
| CT quantitative model | 0.750 (0.677-0.824) | 0.695 | 0.720 | 0.671 | 0.678 | 0.713 | 0.236 |
| Combined model | 0.782 (0.713-0.852) | 0.737 | 0.671 | 0.800 | 0.764 | 0.716 | **/** |

*^*^* DeLong test between combinational model and other three models.

A *P* value less than 0.05 was considered statistically significant.

AUC, area under the curve; CI, confidence interval; ACC, accuracy; SEN, sensitivity; SPE, specificity; PPV, positive predictive value; NPV, negative predictive value; V/M, vessels that encapsulate tumor clusters (VETC) and/or microvascular invasion (MVI). PSM, propensity score matching.

**Figure S1.** Calibration curves of the combined model-based nomogram for predicting V/M+ HCC in the training cohort (a) and external validation cohort (b). The y-axis displays the actual V/M+ HCC rate, while the x-axis illustrates the predicted V/M+ HCC possibility.

b


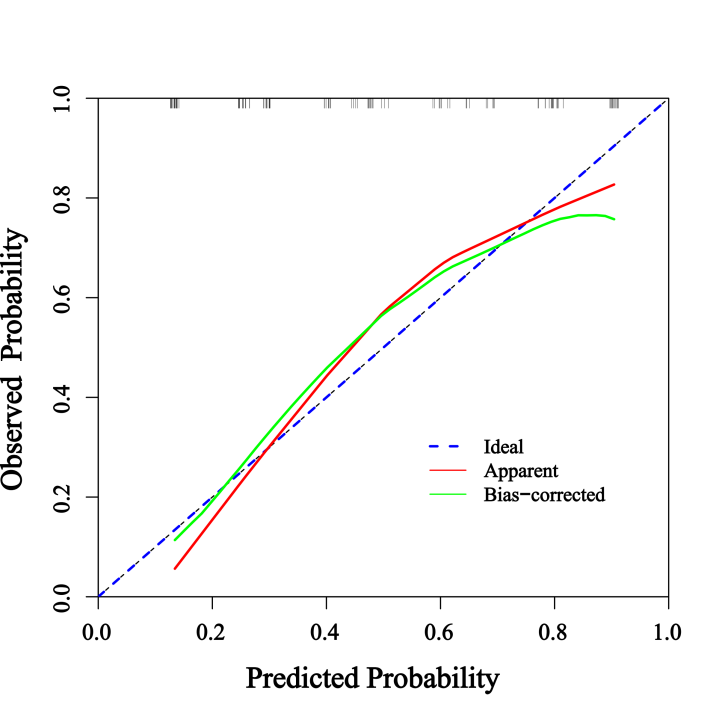

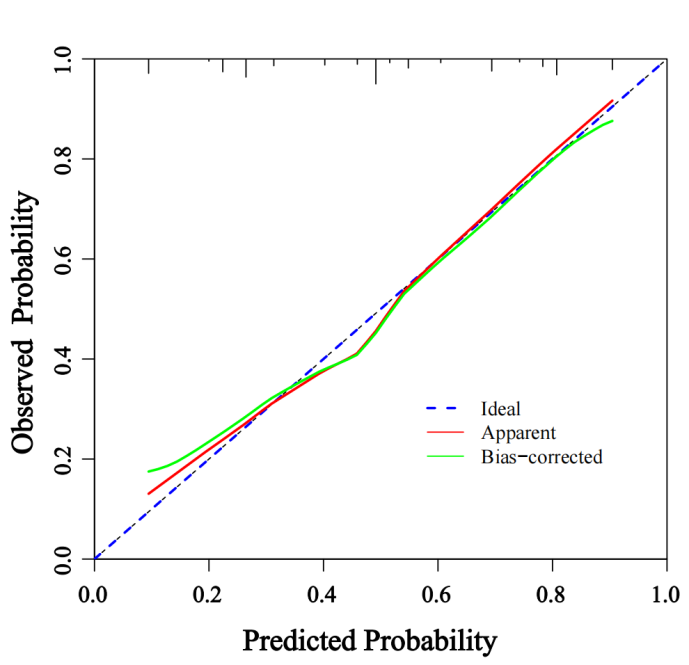


a

**Figure S2.** Decision curve analysis for the combined model-based nomogram in the training cohort (a) and external validation cohort (b). The red and green lines display the impact of two strategies: “treat all patients” and “treat none patients”, respectively. The blue line represents the net benefit of the nomogram at different threshold probabilities.


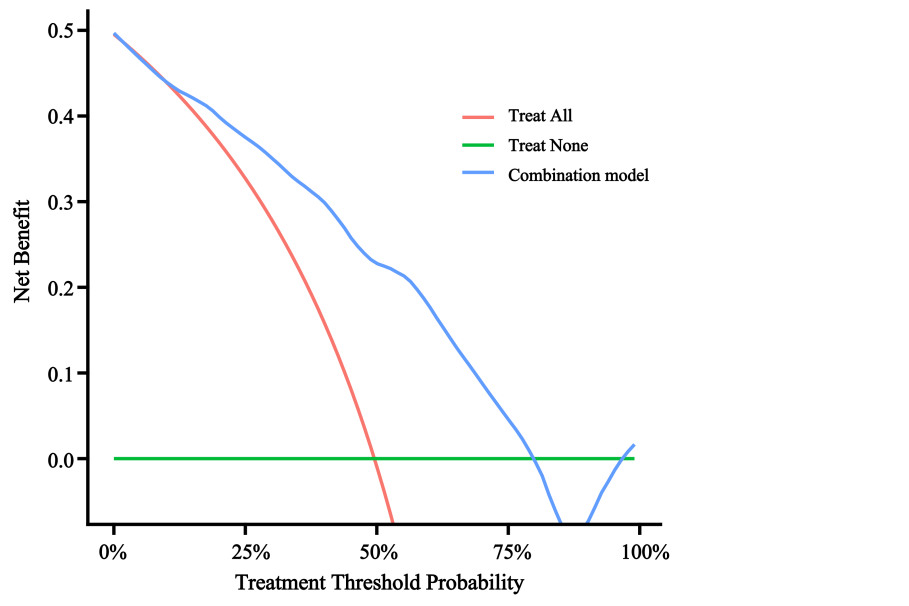

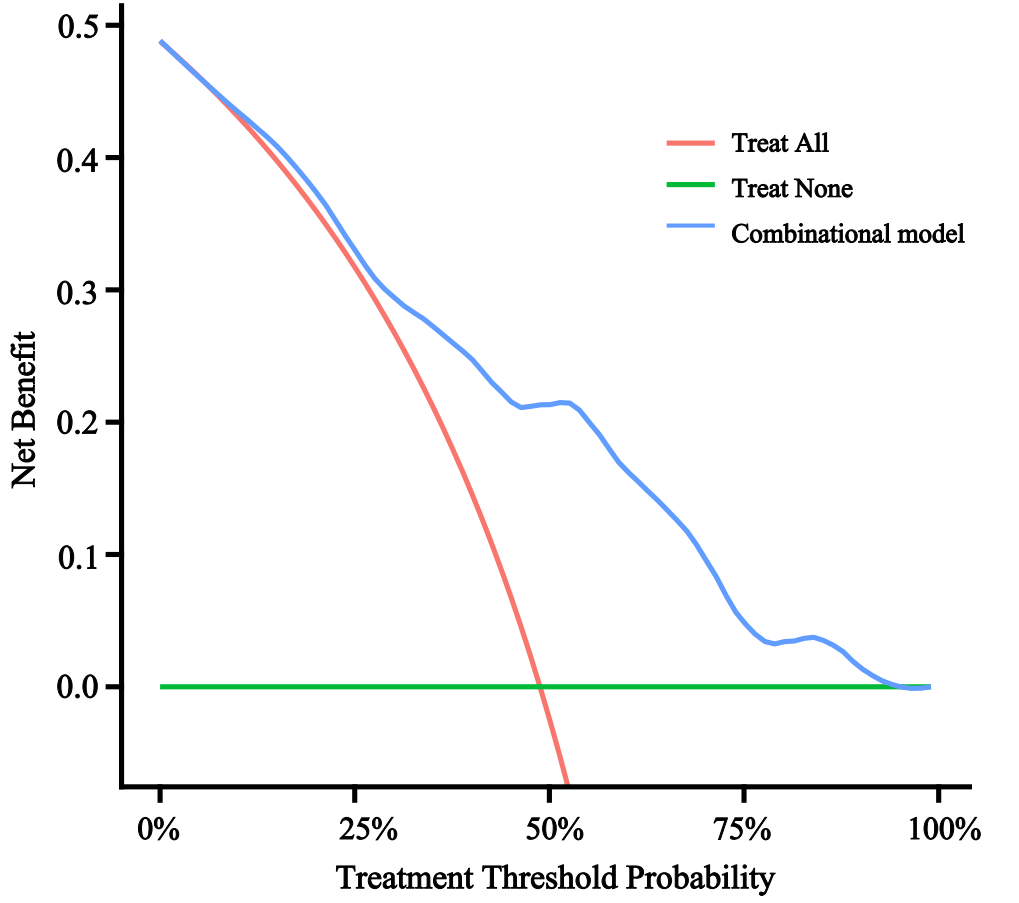


a

b


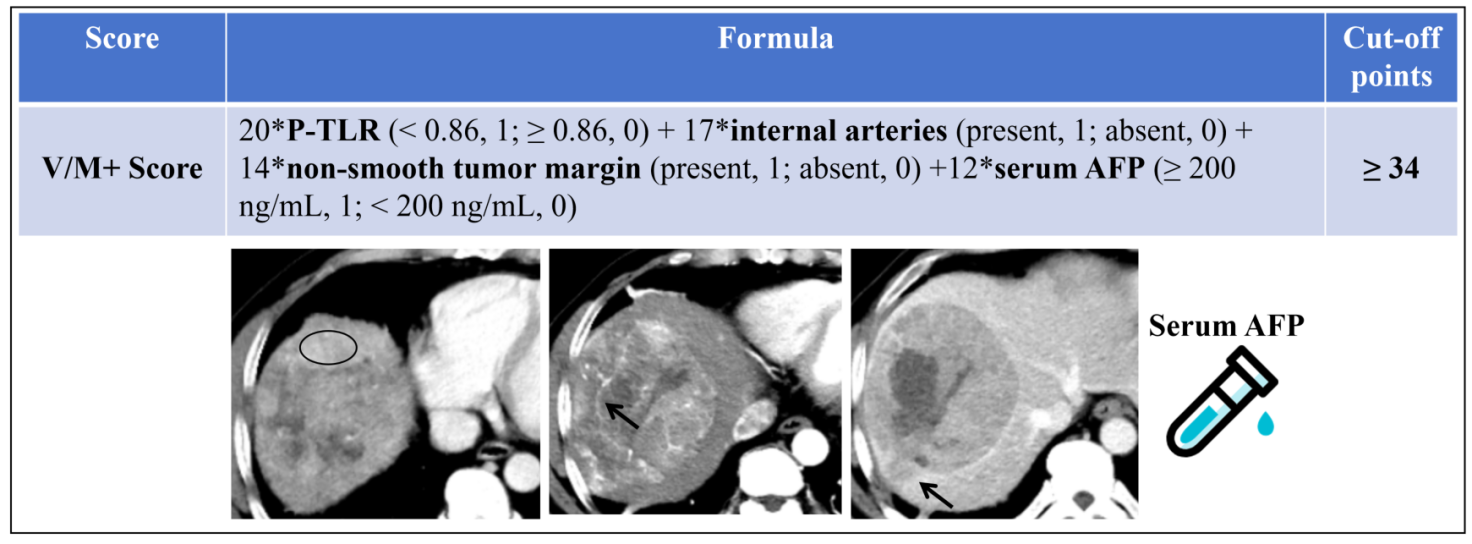


**Figure S3.** A schematic illustration of V/M+ score. V/M, vessels that encapsulate tumor clusters (VETC) and/or microvascular invasion (MVI); P-TLR, tumor-to-liver density ratio in portal venous phase; AFP, alpha-fetoprotein.

**Figure S4.** Kaplan-Meier curves of tumor recurrence-free survival outcomes stratified by V/M+ score (a) and pathologically confirm V/M status (b) after sensitivity analysis by using PSM in the training cohort. Statistical comparison between survival curves was performed with the log-rank test. RFS, recurrence-free survival; PSM, propensity score matching; V/M, vessels that encapsulate tumor clusters (VETC) and/or microvascular invasion (MVI).

a

b


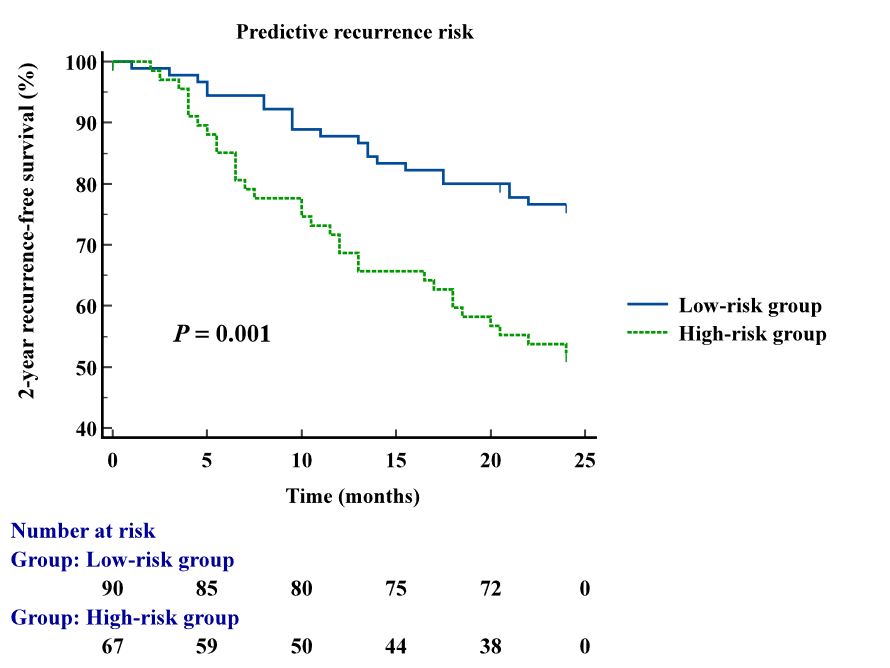

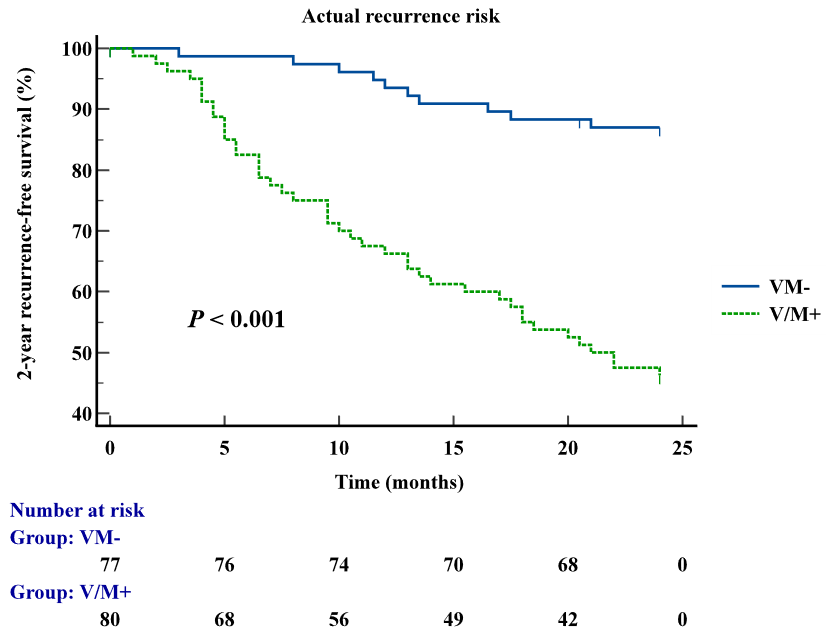

Supplement: Supplementary file 1 — Supplementary information [file 13244_2026_2224_MOESM1_ESM.docx]
